# Functional genomics of pH homeostasis in *Corynebacterium glutamicum *revealed novel links between pH response, oxidative stress, iron homeostasis and methionine synthesis

**DOI:** 10.1186/1471-2164-10-621

**Published:** 2009-12-21

**Authors:** Martin Follmann, Ines Ochrombel, Reinhard Krämer, Christian Trötschel, Ansgar Poetsch, Christian Rückert, Andrea Hüser, Marcus Persicke, Dominic Seiferling, Jörn Kalinowski, Kay Marin

**Affiliations:** 1 University of Cologne, Institute of Biochemistry, Zuelpicher Str. 47, 50674 Cologne, Germany; 2 Ruhr-University of Bochum, Plant Biochemistry, Universitaetsstrasse 150, 44801 Bochum, Germany; 3 Institute for Genome Research and Systems Biology at the Centre for Biotechnology (CeBiTec), Universitaetsstr. 27, 33615, Bielefeld, Gemany; 4 University of Bielefeld, Department of Genetics, Universitaetsstr. 25, 33615, Bielefeld, Germany

## Abstract

**Background:**

The maintenance of internal pH in bacterial cells is challenged by natural stress conditions, during host infection or in biotechnological production processes. Comprehensive transcriptomic and proteomic analyses has been conducted in several bacterial model systems, yet questions remain as to the mechanisms of pH homeostasis.

**Results:**

Here we present the comprehensive analysis of pH homeostasis in *C. glutamicum*, a bacterium of industrial importance. At pH values between 6 and 9 effective maintenance of the internal pH at 7.5 ± 0.5 pH units was found. By DNA microarray analyses differential mRNA patterns were identified. The expression profiles were validated and extended by 1D-LC-ESI-MS/MS based quantification of soluble and membrane proteins. Regulators involved were identified and thereby participation of numerous signaling modules in pH response was found. The functional analysis revealed for the first time the occurrence of oxidative stress in *C. glutamicum *cells at neutral and low pH conditions accompanied by activation of the iron starvation response. Intracellular metabolite pool analysis unraveled inhibition of the TCA and other pathways at low pH. Methionine and cysteine synthesis were found to be activated *via *the McbR regulator, cysteine accumulation was observed and addition of cysteine was shown to be toxic under acidic conditions.

**Conclusions:**

Novel limitations for *C. glutamicum *at non-optimal pH values were identified by a comprehensive analysis on the level of the transcriptome, proteome, and metabolome indicating a functional link between pH acclimatization, oxidative stress, iron homeostasis, and metabolic alterations. The results offer new insights into bacterial stress physiology and new starting points for bacterial strain design or pathogen defense.

## Background

Bacteria have to cope with changing environmental conditions in order to survive in different habitats. A key determinant is the pH value because it has an impact on the solubility of nutrients and trace elements, like iron, and on the cellular metabolism in general. Most bacteria maintain a neutral or slightly alkaline internal pH when subjected to acidic or alkaline conditions [1]. This pH homeostasis is important for the function of all cellular enzymes as well as their stability. The pH gradient across the membrane (ΔpH) can be very high at low pH values or can even be reversed at high external pH values. Beside the electrical membrane potential ΔΨ, ΔpH represents the chemical constituent of the proton motive force (pmf) which is essential for generation of ATP by the F_1_F_0_ATPase.

*Corynebacterium glutamicum *is a work horse in biotechnology for the production of glutamate and lysine and a model strain for the investigation of its pathogenic relatives *C. diphtheriae*, *C. jeikeium *or mycobacteria [2-4]. Its sensitivity towards acidic pH was noticed, but regarding the mechanism of pH homeostasis and the components participating in the acclimatization process, little is known.

Several general mechanisms are known to be important during pH acclimatization in bacteria. Under alkaline conditions, sodium proton antiporters like MdfA and NhaA mediate resistance in *E. coli *[5,6]. However, in *C. glutamicum *an MdfA homologue is missing and the participation of further sodium proton antiporters in the pH response is unknown. Arginine, lysine, and glutamate decarboxylases are predominant for acid tolerance in many bacteria. During decarboxylation of amino acids CO_2 _is liberated and affects the internal pH by formation of bicarbonate. The decarboxylated product is excreted in exchange for the corresponding amino acid [7]. In *C. glutamicum *genes encoding homologous proteins of the AdiCA (arginine:agmatine antiporter and arginine decarboxylases), GadABC (glutamate decarboxylase AB and glutamate:gamma-aminobutyric acid antiporter), and CadAB (lysine decarboxylases and lysine:cadaverine antiporter) systems are absent [8]. In Gram-positive bacteria like *Bacillus subtilis*, or lactic acid bacteria the arginine deiminase pathway is important for acid stress response [7]. By arginine utilization ammonium is liberated which induces the alkalization of the cytoplasm as well as the periplasm. In *C. glutamicum*, however, a homolog of the *arcA *gene is missing (Kalinowski *et al*., 2003). The F_1_F_0_ATPase was found to function as a proton exporter under acidic conditions in *Enterococcus hirae *and its role in pH homeostasis in other bacteria was discussed [7,9]. In *C. glutamicum *the *atp *gene cluster encoding the F_1_F_0_ATPase was found to be transcriptionally induced at alkaline pH under the control of the sigma factor SigH and subsequent studies indicated that the expression is correlated with growth rate rather than the pH value of the medium [10,11]. Furthermore a putative cobalt transporter encoded by the gene *cg1447 *was found to be important under alkaline conditions [12].

Further studies on acidic pH response revealed the participation of multiple cellular processes in acclimatization of various bacteria. Among them are the activation of the protein folding and stabilization machinery [13], the induction of iron uptake systems [11], or metabolic adaptations including the induction of the methionine pathways [14]. Furthermore, observations were made indicating the occurrence of oxidative stress at low pH values [15]. In conclusion, a shift of the external pH seems to act on various levels and affects a multiplicity of cellular processes finally limiting growth at non optimal pH conditions.

In the present study we identified limitations of pH homeostasis that restrict growth at non-optimal pH conditions in *C. glutamicum*. We excluded short term effects and focused on the steady state regulation in exponentially growing cells under neutral, acidic, and alkaline conditions. Applying transcriptome studies, soluble as well as membrane proteome analyses we found that *C. glutamicum *cells are exposed to oxidative stress at low pH and concomitantly iron starvation response is induced leading to the alteration of a variety of metabolic pathways which was reflected by differential metabolite pattern as well. We present comprehensive data showing that a decrease of the external pH affects particular cellular processes at various levels which finally limit growth of *C. glutamicum *under acidic conditions.

## Results

### Effective pH homeostasis is correlated with optimal growth in *C. glutamicum*

**Figure 1:**
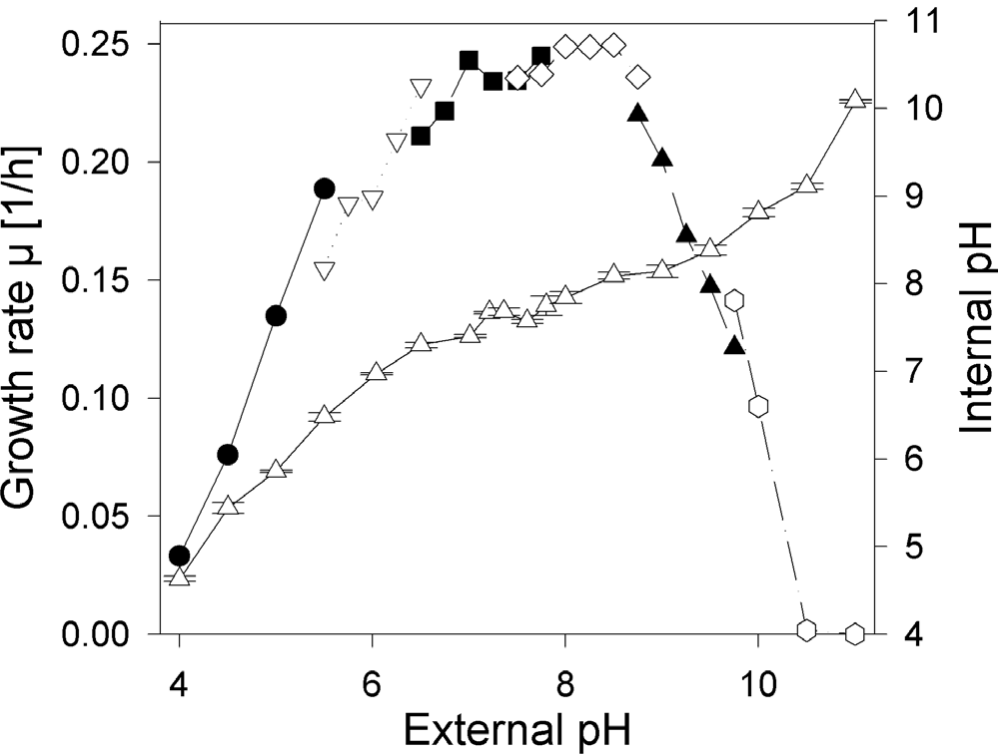
**Comparison of growth rate and internal pH of *C. glutamicum *exposed to different external pH values**. Growth experiments were performed in selected buffer systems in shaken microtiter plates (pH 4-5.5 black circles: Homopipes, pH 5.5-6.5 white triangles down: Mes, pH 6.5-7.75 black squares: Mops, pH 7.5-8.75 white diamonds: Hepps, pH 8.75-9.75 black triangles: Ches, pH 9.75-11 white hexagons: Caps). Determination of the cytoplasmic pH (white triangles top) was performed in a separate experiment by measuring the distribution of the radioactive probes benzoic acid (pH 4 to 7.5) and methylammonium (pH 7.5-11, see Methods section).

We first quantified the efficiency of pH homeostasis in *C. glutamicum*. We performed growth assays in shaken micro titer plates (MTP) in minimal medium in presence of optimized buffers at a pH of 4 to 11 with subsequent determination of growth rates. As seen in Fig. 1, optimal growth rates were observed at a pH of 7 to 8.5. At an external pH below 6 and above 9 growth rates decreased drastically and at a pH of 4 as well as 10.5 and 11 no significant growth was observed.

Subsequently, we determined the internal pH of *C. glutamicum *cells grown at pH 7.5 after exposure to different external pH values. At a pH of 7.5 the internal pH value was found to be 7.5. This value was kept constant (± 0.5 pH units) after lowering the external pH down to 6 or increasing the pH up to 9. Below or above these external pH values the internal pH decreased respectively increased much faster in response to an external pH shift. We concluded that *C. glutamicum *can perform effective pH homeostasis in a range of external pH values from 6 to 9.

**Figure 2:**
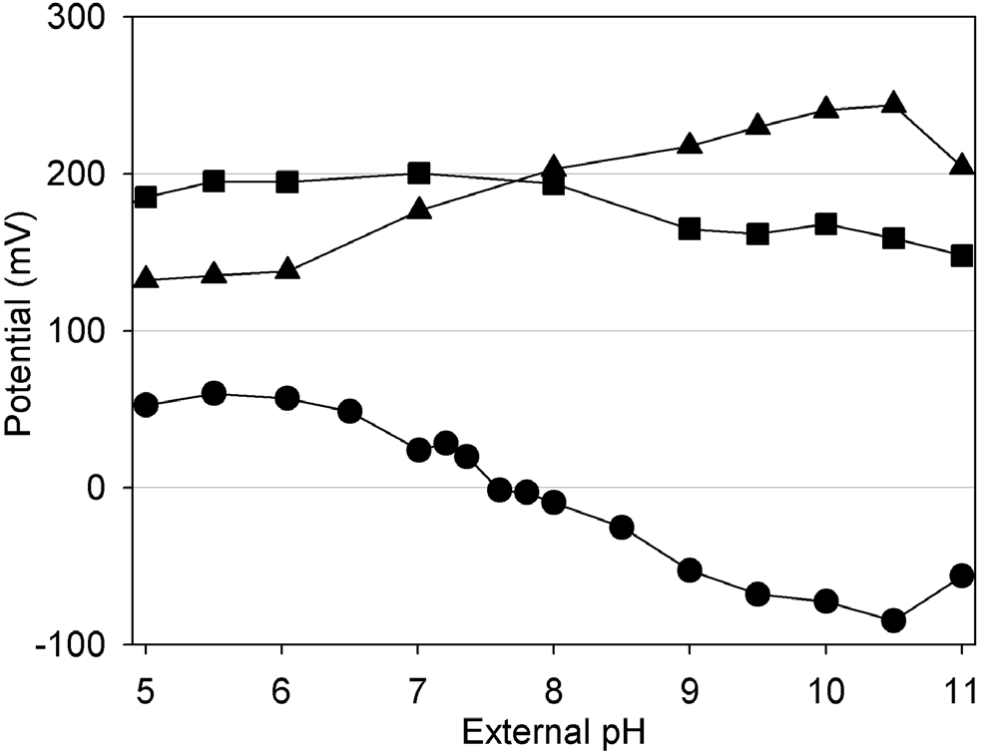
**The pH dependent bioenergetic homeostasis in *C. glutamicum***. Membrane potential (triangles) and pH gradient (circles) across the cytoplasmic membrane of *C. glutamicum *exposed to different external pH values and values for the resulting proton motive force (squares). All values are given in mV.

The failure of effective pH homeostasis at low or high external pH values could result from an impaired energy metabolism. The pH gradient across the cytoplasmic membrane (ΔpH) is important for generation of the proton motive force (pmf = ΔΨ - 2.3RT/F × ΔpH) which is essential for ATP synthesis by the F_1_F_0_ATPase. In order to prove whether the pmf is affected in *C. glutamicum *we determined ΔpH as well as the membrane potential ΔΨ in cells exposed to different pH values and calculated the pmf. The results are shown in Fig. 2. As expected, the pH gradient is zero at pH 7.5. At lower external pH values ΔpH increased up to 60 mV, whereas at higher external pH values, ΔpH was found to be reverted and decreased to -80 mV. The values for ΔΨ were found to be 110 mV at an external pH of 4.5, increased to 200 mV at pH 8, and at very high pH values (pH 10.5) 245 mV were measured. As a consequence of the decreasing ΔpH and increasing ΔΨ values the resulting pmf was kept relatively constant at a surprisingly broad pH range of 4.5 to pH 11, varying between 150 to 200 mV. At the most acidic pH of 4 the membrane potential ΔΨ collapsed and the resulting pmf value was 40 mV only.

### Transcriptome and proteome analyses of pH acclimatization in *C. glutamicum*

In order to unravel components and processes involved in pH homeostasis we performed transcriptome analyses by DNA microarrays and proteome studies by 1D-nLC-ESI-MS/MS. For this purpose, two independent batch fermentations were carried out at a pH of 6, 7.5, and 9 in stirred bioreactors under continuous pH control and samples were drawn during the exponential phase in order to focus on the steady state pH homeostasis and to prevent additional perturbations by short term responses. We observed growth rates of 0.14 ± 0.01 at pH 6, 0.32 ± 0.02 at pH 7.5, and 0.14 ± 0.01 at pH 9. Cells were harvested and immediately frozen for metabolic inactivation. Transcriptome patterns were analyzed by co-hybridization of cDNA derived from cells grown at pH 6 vs. pH 7.5 and from cells grown at pH 9 vs. pH 7.5. The data analysis was performed as described previously [16], using a m-value (log_2 _of the relative change in the respective mRNA ratio) cut-off of ± 1 which corresponds to transcription changes equal or greater than twofold. For the comparative proteome analysis we performed identification and quantification of peptides in the enriched soluble and membrane fractions as well as in the cell envelope fraction. Relative quantification of protein abundance and its change was performed by the spectral counting technique and the results are also given as log_2 _values [17]. The complete set of data is available as supplementary material.

The transcriptome analyses revealed 42 genes with increased expression at pH 9 in comparison to pH 7.5 (Table 1). In the respective proteome studies, 19 corresponding proteins were found with an increased peptide number in at least one protein fraction whereby four of them were present at significantly higher levels at pH 9 in comparison to pH 7.5 (Table 1). For 39 genes we found a decreased mRNA level at pH 9 in comparison to pH 7.5 whereby for 26 corresponding proteins a lower content was indicated by lower peptide numbers in at least one fraction and for four of them a significant lower abundance at pH 9 was observed (Table 1). For 10 genes differentially expressed at pH 9 we did not find a corresponding change of the peptide number and for 18 proteins we did not find any corresponding peptides at all indicating a low abundance of these proteins in *C. glutamicum *cells. The comparison of cells grown at pH 6 and pH 7.5 revealed higher mRNA pools for 88 genes whereby for 49 corresponding proteins (for 10 of them significantly) increased peptide numbers were found (Table 2). A lower mRNA content at pH 6 was found for 91 genes whereby for 52 corresponding proteins (for 16 of them significantly) decreased peptide numbers were found at least in one protein fraction at acidic pH (Table 2). In case of 17 proteins alterations of mRNA and protein content do not match at acidic pH. For 35 genes differentially expressed at pH 6 in comparison to pH 7.5 no peptide was found at all. In summary, we found many overlaps of transcriptome and proteome data for *C. glutamicum *grown at different pH values.

**Table 1:** Differential expression pattern at pH 9 in comparison to pH 7.5

Genes induced at pH 9						Transcriptome^4^		Proteome^5^									Regulators^6^
								cytoplasma			envelope			membrane			
No	gene ID^1^	op^2^	gene	function	TMH^3^	pH 6	pH 9	6	7.5	9	6	7.5	9	6	7.5	9	
1	*cg0077*			Hypothetical protein	0	n.d.	1.04	-0.6	-	-	-	-	-	-0.9	-	-	
2	*cg0105*			Hypothetical protein Cgl0077	0	-0.83	1.65	-	-	-	-0.4	-	-	-	-	-	
3	*cg0310*		*katA*	Catalase	0	-0.62	1.73	4.2	5.1	6.4	-	-	0.0	-	-	-0.6	RipA
4	*cg0444*		*ramB*	Regulator of acetate metabolism	0	0.76	1.11	-	-	-0.8	-	0.7	3.5	-	-	-	RamA, RamB
5	*cg0445*	c	*sdhCD*	Succinate dehydrogenase component CD	5	-1.31	1.31	-	-	-	1.8	1.4	2.4	3.2	4.0	5.0	RipA, DtxR, RamA*, RamB*
6	*cg0446*	c	*sdhA*	Succinate dehydrogenase A	0	-1.08	1.47	1.0	2.7	1.7	6.7	7.0	***9.0***	***8.5***	9.0	***9.9***	RipA, DtxR, RamA*, RamB*
7	*cg0447*	c	*sdhB*	Succinate dehydrogenase B	0	-1.43	1.14	-	-0.4	-	4.6	5.0	***6.7***	3.5	3.4	***5.3***	RipA, DtxR, RamA*, RamB*
8	*cg0448*	c		Conserved hypothetical membrane protein	2	-0.98	1.18	-	-	-	-0.4	0.8	1.7	-	-	-	RipA*, DtxR, RamB*
9	*cg0778*		*fecC*	putative iron or siderophore ABC-type transporter, permease component	8	-0.05	1.07	-	-	-	-	-	-	-	-	-	
10	*cg0825*			Dehydrogenases with different specificities	0	-0.19	1.07	4.1	4.6	5.0	-0.4	-0.3	1.8	-0.7	-	-0.4	
11	*cg0858*			putative gamma subunit of the nitrate reductase	1	-0.28	1.22	-	-	-	-	-	-	-	-	-	
12	*cg1095*			Hypothetical protein	0	0.32	1.28	-	-	-	-	-	-	-	-	-	
13	*cg1136*			Hypothetical protein	0	0.15	1.02	1.6	0.5	2.2	-	-	-	-	-	-	
14	*cg1206*			PEP phosphonomutase or related enzyme	0	0.38	1.05	2.6	2.8	2.7	-	-	-	-	-	-	
15	*cg1292*	r		Flavin-containing monoxygenase 3	0	n.d.	3.87	-	-	2.4	-	-	4.4	-	-	-0.6	
16	*cg1293*	r		Hypothetical membrane protein	3	n.d.	1.71	-	-	-	-	-	-	-	-	-	
17	*cg1312*			Hypothetical membrane protein	4	n.d.	1.32	-	-	-0.2	1.9	2.1	***4.7***	5.3	4.4	***6.3***	
18	*cg1344*	s	*narG*	Nitrate dehydrogenases 2 (Fe4S4 containing)	0	1.36	1.94	-	-	-0.6	-	-	-	-	-	-0.4	RipA, GlxR*
19	*cg1345*	s	*narK*	putative nitrate/nitrite transporter	12	n.d.	1.66	-	-	-	-0.4	-	3.0	1.5	-0.6	3.0	RipA, GlxR*
20	*cg1695*			SAM dependent methyltransferase	0	n.d.	1.72	-	-	-	-	-	-	-	-	-	
21	*cg1737*		*acn*	Aconitase A	0	-0.58	1.66	4.4	5.3	***6.2***	4.4	5.0	4.9	2.2	2.6	2.5	RipA, RamA*, RamB*
22	*cg1759*	x	*sufX*	Predicted metal-sulfur cluster biosynthetic enzyme	0	0.94	1.34	-	-	-0.8	-0.4	-	-	-	-	-	SufR, SigM
23	*cg1761*	x	*nifS2*	Cysteine desulfhydrase, Selenocysteine lyase	0	0.44	1.47	2.3	2.0	3.2	-0.4	-	-	-	-	-	SufR, SigM
24	*cg1762*	x	*sufC*	Suf related ABC-type transporter, ATPase component	0	0.81	1.51	3.7	3.4	4.6	3.4	4.4	4.1	0.1	2.9	2.1	SufR, SigM
25	*cg1764*	x	*sufB*	Suf related ABC-type transporter SufB, permease component	0	0.93	1.81	2.8	3.4	5.0	3.4	3.4	3.8	0.1	0.4	1.6	SufR, SigM
26	*cg1765*	x	*sufR*	transcriptional regulator SufR	0	0.76	1.53	-	-	-	-	-	-	-	-	-	SufR, SigM
27	*cg1790*		*pgk*	Phosphoglycerate kinase	0	0.15	1.07	4.8	4.2	5.0	5.0	4.8	5.0	2.5	2.7	3.3	SigB*
28	*cg1884*			putative copper export protein CopC	2	-0.39	1.12	-	-	-	-	-	-	-	-	-	LexA
29	*cg1904*			ABC-type transporter, permease component	6	0.09	1.12	-	-	-	-	-	-	-	-0.6	1.2	
30	*cg2191*			putative 3-demethylubiquinone-9 3-methyltransferase	0	n.d.	1.02	-	-	-	-	-	-	-	-	-	
31	*cg2274*	ab		Hypothetical protein	0	-0.17	1.15	-0.6	0.4	1.8	-	-	-	-	-	-	
32	*cg2275*	ab		Hypothetical protein	0	-0.28	1.03	1.0	2.4	1.3	-	-	-	-0.9	-	-	
33	*cg2320*			ArsR type transcriptional regulator	0	-0.26	1.25	-	-	-	-	-	-	-	-	-	
34	*cg2572*			Hypothetical protein	0	0.45	1.44	-	-	-	-	-	-	-	-	-	
35	*cg2636*		*catA1*	Protocatechuate 3,4-dioxygenase beta subunit	0	n.d.	3.09	-	-	1.6	-	-	-	-	-	-	RipA, GlxR*
36	*cg2736*		*bcp*	putative 3-demethylubiquinone-9 3-methyltransferase	0	0.12	1.12	0.5	-0.5	0.4	-	-	-	-	-	-	
37	*cg2782*		*ftn*	Ferritin-like protein	0	0.56	1.29	-	-	0.8	-	-	-	-	-	-	DtxR
38	*cg2853*			Hypothetical protein	0	0.41	1.4	0.6	-0.5	1.8	-	-	-	-	-	-	
39	*cg3117*	ai	*cysX*	Hypothetical protein	0	3.5	1.08	-	-	-	-	-	-	-	-	-	DtxR, McbR*, CysR*
40	*cg3118*	ai	*cysI*	Sulfite reductase hemoprotein beta-component	0	3.5	1.06	0.6	1.5	2.4	3.5	2.1	-0.3	-	-	-	DtxR, McbR, CysR*
41	*cg3236*			MFS-type transporter	0	1.27	1.01	-	-	-0.8	-	-	-	-	-	-	
42	*cg3331*		*ogt*	Methylated DNA-protein cysteine methyltransferase	0	0.2	1.34	-	-0.8	-	-0.1	-	1.3	-	-	-	
**Genes repressed at pH 9**																	

1	*cg0071*			Metallo-beta-lactamase superfamily	0	n.d.	-1.01	-	-	-	-	-	-	-	-	-	
2	*cg0133*		*abgT*	P-aminobenzoyl-glutamate transporter	13	-0.86	-1.02	-	-	-	-	-	-	0.3	3.7	2.1	
3	*cg0202*		*iolD*	Putative acetolactate synthase	0	n.d.	-1.13	-	-	-	-	-	-	-	-	-	
4	*cg0265*			putative ABC-type molybdate transporter, ATPase component	0	-0.22	-1.08	-	-	-	-	-	-	-	-	-	
5	*cg0303*		*leuA*	2-isopropylmalate synthase	0	-0.97	-1.06	4.3	4.7	4.5	-	-0.3	-	-	-	-	
6	*cg0404*	b		putative nitroreductase	0	1.77	-1.50	3.4	2.5	0.9	0.9	-	-	-0.9	-	-	
7	*cg0467*			ABC-type cobalamin/Fe3+-siderophores transporter, periplasmic component	0	-0.50	-1.10	-	0.2	-	-	0.7	-	0.2	1.0	-0.4	DtxR
8	*cg0527*			ArsR type transcriptional regulator	0	-0.32	-1.13	-	-	-	-	-	-	-	-	-	DtxR
9	*cg0589*			ABC-type cobalamin/Fe3+-siderophores transporter, ATPase component	0	-0.15	-1.01	-	-	-	1.2	-	-	0.1	-	-	DtxR
10	*cg0623*			putative ABC-type cobalt transporter, permease components	8	-1.05	-1.35	-	-	-	-	-	-	-	-	-	
11	*cg0624*	h		Hypothetical membrane protein	2	-1.09	-1.09	-	-	-	-	-	-	1.3	1.6	-0.4	
12	*cg0625*	h		putative terpenoide cylase	1	-1.28	-1.04	-	2.2	0.0	2.8	3.6	2.2	-	-0.6	-0.4	
13	*cg0723*		*crtE*	Geranylgeranyl-pyrophosphate sythase	0	0.66	-1.01	-	-	-	-	-0.2	-	-	-	-	
14	*cg0748*			ABC-type Fe3+-siderophores transporter, periplasmic components	0	-1.07	-2.14	-0.3	1.3	-	1.2	0.7	-	0.1	-0.6	-	DtxR
15	*cg0756*		*cstA*	putative carbon starvation protein A	16	-0.83	-1.56	-	-	-	1.9	2.1	1.0	2.1	2.8	-0.6	
16	*cg0767*	m		Siderophore-interacting protein	0	-0.34	-2.99	0.5	-0.8	-	-	-	-	-	-	-	DtxR, RamB*
17	*cg0768*	m		ABC-type cobalamin/Fe3+-siderophores transporter, ATPase component	0	-0.17	-2.03	-	-	-	1.7	1.7	-	-	-0.6	-	DtxR, RamB*
18	*cg0924*	n		ABC-type cobalamin/Fe3+-siderophores transporter, periplasmic component	0	-0.60	-2.91	4.8	5.2	**2.7**	3.6	4.1	0.7	5.1	4.5	3.4	DtxR
19	*cg0927*	n		ABC-type cobalamin/Fe3+-siderophores transporter, permease component	9	-0.10	-2.08	-	-	-	1.2	-0.2	0.0	3.0	3.3	-	DtxR
20	*cg0928*	n		ABC-type cobalamin/Fe3+-siderophores transporter, ATPase component	0	-0.14	-2.35	-	-0.8	-	3.3	4.0	-	1.8	1.0	-	DtxR
21	*cg0952*	o	*mctB*	putative monocarboxylate transporter subunit	2	-2.55	-1.32	-	0.5	-	2.4	4.7	4.1	-	2.1	-0.6	RamA, RamB
22	*cg0953*	o	*mctC*	monocarboxylate transporter	13	-2.44	-1.01	-	3.0	-0.2	1.9	4.7	4.7	3.2	4.5	3.2	RamA, RamB
23	*cg1091*			Hypothetical protein	0	0.95	-1.18	-	-	-	-	-	-	-	-	-	SigM*
24	*cg1167*		*metS*	putative methionine transporter subunit	0	-1.04	-1.43	-	-	-	-	-	-	-0.9	-	-0.6	
25	*cg1290*		*metE*	5-methyltetrahydropteroyltri-glutamate-homocystein-emethyltransferase	0	0.65	-1.06	8.2	6.8	6.4	8.0	8.0	**6.5**	6.2	6.4	**4.2**	McbR*
26	*cg1365*	t	*atpH*	F0F1 ATP synthase delta subunit	0	-1.26	-1.03	1.9	2.3	1.3	3.9	4.8	4.7	3.2	4.1	3.4	SigH*
27	*cg1367*	t	*atpG*	F0F1 ATP synthase gamma subunit	0	-1.71	-1.11	1.1	4.2	3.4	3.3	4.9	4.7	4.0	4.0	2.5	SigH*
28	*cg1451*		*serA*	Phosphoglycerate dehydrogenase	0	-0.22	-1.10	**6.5**	5.4	**6.1**	7.7	7.5	7.2	5.3	5.3	**4.2**	
29	*cg1537*		*ptsM*	PTS system mannose-specific EIIBCA component	10	-0.39	-1.06	2.6	3.5	1.8	5.9	5.6	6.0	6.6	6.4	6.1	RamB*, GlxR*
30	*cg1859*			Putative secreted protein	0	0.04	-1.34	-	1.7	-	-	3.6	1.5	0.1	5.0	-0.4	
31	*cg1930*	z		Putative secreted hydrolase	0	-1.18	-3.26	-	-	-	-	-	-	-	-	-	DtxR
32	*cg1931*	z		Hypothetical protein	0	-0.88	-2.55	-	-	-	-	-	-	-0.7	-0.6	-	DtxR
33	*cg2283*			Hypothetical protein	0	0.00	-1.51	4.2	4.3	3.4	-	-0.2	-	-	-	-	
34	*cg2336*			Putative secreted protein	0	-0.09	-1.31	-	-	-	-	2.1	-	3.6	3.9	1.9	
35	*cg2445*		*hmuO*	putative heme oxygenase	0	-0.93	-1.19	-0.2	2.5	-	-	-0.2	-	-	-0.6	-	DtxR
36	*cg2560*	ad	*aceA*	Isocitrate lyase	0	-2.73	-2.19	-	4.9	**2.4**	-	-0.3	-	-	-	-	RamA, RamB
37	*cg2962*			Uncharacterized enzyme involved in biosynthesis of extracellular polysaccharides	0	0.45	-1.18	-0.3	0.2	-	-	-	-	-	-	-	
38	*cg3156*		*htaA*	secreted protein implicated in iron acquisition and transport	0	-2.50	-2.76	-	-	-	-	-	-0.3	2.7	5.7	-	DtxR
39	*cg3254*			Hypothetical membrane protein	3	0.07	-1.29	-	-	-	-	-	-	-0.5	-	-	

Genes for which an increased or decreased mRNA level was found at pH 9 in comparison to pH 7.5. The gene locus tag, organisation in operons, the gene name, the (proposed) function of the protein as well as the predicted number of transmembrane helices are given. The results of the transcriptome analysis are given as induction factor at pH 6 and pH 9 in comparison to pH 7.5. Results of the proteome analysis are indicated for the soluble, membrane and envelope fraction. Regulators of particular genes are given based on the CoryneRegNet data base.

^1 ^The geneID according to the accession number BX927147 was used.

^2 ^Genes known to form an operon and closely adjacent, equally oriented genes that likely form an operon were indicated by equal Latin letters.

^3 ^Prediction of transmembrane helices were performed by using the TMHMM 2.0 sever at http://www.cbs.dtu.dk/services/TMHMM/.

^4 ^The induction factors are given as log_2 _values of the ration of mRNA levels at pH 6 and pH 9 in comparison to pH 7.5, respectively.

^5 ^The determined relative peptide numbers are given as log_2 _values in order to allow calculation of ratios by simple subtraction of values. Peptide numbers found to be significantly altered at pH 6 and pH 9 in comparison to pH 7.5 are shown in bold and peptide numbers found to be significantly altered at pH 6 in comparison to pH 9 are shown in italic (see M&M section for the details of calculation).

^6 ^Data whether a particular gene was experimentally proven or predicted (*) to be regulated by a transcription factor was obtained by using the data base CoryneRegNet http://coryneregnet.cebitec.uni-bielefeld.de/v4/.

**Table 2:** Differential expression pattern at pH 6 in comparison to pH 7.5

Genes induced at pH 6						Transcriptome^4^		Proteome^5^									Regulators^6^
								cytoplasm			envelope			membrane			
No	gene ID^1^	op^2^	gene	function	TMH^3^	pH 6	pH 9	6	7.5	9	6	7.5	9	6	7.5	9	
1	*cg0012*			Hypothetical protein	0	1.45	n.d.	-	-	-	2.5	-	-	-	-	-	McbR*
2	*cg0325*			Multisubunit Na+/H+ antiporter	2	1.04	0.17	-	-	-	0.6	1.3	0.8	-	-	-	
3	*cg0360*			Putative phosphatase	1	1.35	n.d.	-	-	-	-	-	-	-	-	-	
4	*cg0403*	b	*rmlB1*	dTDP-glucose 4,6-dehydratase	0	1.04	n.d.	-	-	-	-	-	-	-	-	-	
5	*cg0404*	b		Nitroreductase family	0	1.77	-1.50	3.4	2.5	0.9	0.9	-	-	-0.9	-	-	
6	*cg0550*			Putative peptidase E	0	1.10	-0.46	-	-	-	-	-	-	-	-	-	
7	*cg0736*	k	*metN*	ATPase component	0	1.45	n.d.	2.2	-	-	***5.1***	2.8	2.6	4.0	1.7	-0.6	McbR*, RamB*
8	*cg0737*	k	*metQ*	periplasmic component	0	1.85	-0.69	***5.4***	3.4	3.5	***7.2***	4.1	4.7	***9.1***	6.8	6.7	McbR*, RamB*
9	*cg0754*	l	*metX*	Homoserine O-acetyltransferase	0	1.19	n.d.	2.1	-	-0.8	2.5	-	-0.3	-	-	-	McbR*
10	*cg0755*	l	*metY*	O-acetylhomoserine sulfhydrylase	0	2.43	-0.08	***6.6***	4.8	5.4	3.3	0.7	1.4	1.7	-	-	McbR*
11	*cg0874*			Uncharacterized ACR, COG2135	0	1.03	0.46	-	-	-	-0.4	-	-	-	-	-	
12	*cg0878*		*whiB1*	Stress response transcription factor WhiB1	0	1.26	0.49	-	-	-	-	-	-	-	-	-	SigH, GlxR*
13	*cg1081*	p		ABC-type multidrug transport system, ATPase component	0	1.18	0.86	-0.3	-0.8	0.0	2.0	2.6	3.3	3.0	3.3	4.4	
14	*cg1082*	p		ABC-type multidrug transporter, permease components	6	1.33	0.68	-	-	-	-	-	1.4	-	-	-	
15	*cg1083*	p	*cgtS10*	Two-component system, sensory transduction histidine kinases	5	1.38	0.64	-	-	-	-	-	1.0	-	-	-	SigB*
16	*cg1129*		*aroG*	Phospho-2-dehydro-3-deoxyheptonate aldolase	0	1.44	n.d.	1.1	1.5	-	2.4	2.6	-	-0.7	1.4	-	
17	*cg1150*			NADPH dependent FMN reductase	0	1.02	n.d.	1.2	-	-	-	-	-	-	-	-	
18	*cg1202*			Hypothetical protein	0	1.23	n.d.	-	-	-	-	-	-	-	-	-	
19	*cg1214*	q	*sufS*	Cysteine desulfurase involved in maturation of Fe-S clusters	0	1.30	-0.37	-0.6	-	-	1.4	-0.3	-	-	-	-	NrtR*
20	*cg1215*	q	*nadC*	Nicotinate-nucleotide pyrophosphorylase	0	1.30	-0.06	5.1	4.2	3.6	1.2	-	-	2.1	-	1.6	NrtR*
21	*cg1216*	q	*nadA*	Quinolinate synthetase A	0	1.77	-0.20	-	0.2	0.7	2.2	2.6	-	-	0.7	-	NrtR*
22	*cg1218*	q	*nrtR*	Regulator NrtR, ADP-ribose pyrophosphatase	0	1.58	-0.24	-	-	-	-	-	-	-	-	-	NrtR*
23	*cg1256*		*dapD*	Tetrahydrodipicolinate N-succinyltransferase	0	1.25	n.d.	2.5	0.4	-	***5.0***	3.6	2.4	-0.9	-	-	
24	*cg1291*			Hypothetical membrane protein	2	2.43	n.d.	-	-	-	0.9	-	-	-	-	-	
25	*cg1322*			Uncharacterized beta barrel protein	0	1.24	-0.62	***6.3***	5.1	***2.7***	-	-	-	2.6	-0.6	-	
26	*cg1337*		*hom*	Homoserine dehydrogenase	0	1.68	0.22	4.4	2.4	3.0	5.4	4.5	4.0	3.1	3.2	1.4	McbR
27	*cg1344*	s	*narG*	Nitrate reductase	0	1.36	1.94	-	-	-0.6	-	-	-	-	-	-0.4	RipA, GlxR*
28	*cg1476*		*thiC*	Thiamine biosynthesis protein	0	1.00	0.62	3.9	3.8	4.1	2.9	2.3	3.6	1.8	1.4	1.0	
29	*cg1478*			Hypothetical protein	0	4.89	n.d.	-0.6	-	-	-	-	-	-	-	-	LexA
30	*cg1580*	v	*argC*	N-acetyl-gamma-glutamyl-phosphate reductase	0	1.65	-0.17	2.9	2.2	2.2	1.3	-0.2	-	-0.9	-	-	ArgR
31	*cg1581*	v	*argJ*	Glutamate N-acetyltransferase	0	2.03	-0.02	5.8	5.0	5.2	2.8	2.1	2.1	-	-	-	ArgR
32	*cg1582*	v	*argB*	Acetylglutamate kinase	0	1.84	n.d.	1.1	-	-0.8	3.1	-	0.0	-	-	-	ArgR
33	*cg1583*	v	*argD*	Acetylornithine aminotransferase	0	1.66	n.d.	1.9	0.9	0.2	-	-	-	-	-	-	ArgR
34	*cg1584*	v	*argF*	Ornithine carbamoyltransferase	0	1.75	-0.12	1.2	0.4	-0.2	-	-	-	-	-	-	ArgR
35	*cg1586*	v	*argG*	Argininosuccinate synthase	0	1.16	0.05	4.1	4.3	4.3	5.4	5.2	5.1	3.1	3.5	2.8	
36	*cg1626*	w		Hypothetical secreted protein	0	2.12	n.d.	-	-	-	-	-	-	-	-	-	
37	*cg1628*	w		Putative hydrolase	0	2.25	n.d.	-	-	-	1.9	-	-	-	-	-	
38	*cg1647*			ABC-type multidrug transporter, permease components	5	1.06	-0.01	-	-	-	-0.4	-	-0.3	-	-	-	AmtR*
39	*cg1701*		*metH*	Homocysteine Methyltransferase	0	2.22	0.12	1.1	-	-	3.7	3.1	0.7	-	-	-0.6	McbR*
40	*cg1739*			GMP synthase-Glutamine amidotransferase domain	0	1.36	n.d.	-	-	-	-	-	-	-	-	-	McbR*
41	*cg1806*		*metK*	S-adenosylmethionine synthetase	0	1.29	-0.17	2.9	2.3	1.1	3.6	2.9	-	-	-	-	McbR
42	*cg1940*			Hypothetical protein	1	1.18	-0.08	-	-	-	-	-	-	-	-	-	
43	*cg2051*			Hypothetical protein	0	1.25	n.d.	-	-	-	-	-	-	-	-	-	
44	*cg2157*		*terC*	putative tellurium exporter TerC	9	1.05	-0.13	-	-	-	2.8	2.4	1.5	1.5	1.0	2.3	
45	*cg2250*			Hypothetical protein	0	1.16	n.d.	-	-	-	0.6	-	-0.3	1.6	-	1.1	
46	*cg2260*		*glnK*	Nitrogen regulatory protein PII	0	1.13	n.d.	1.2	2.2	1.4	-	-	-	-	-	-	AmtR
47	*cg2338*		*dnaE1*	DNA polymerase III, alpha chain	0	1.12	0.29	-	-	-	0.0	-	-0.3	-	-	-	
48	*cg2380*			Hypothetical membrane protein	2	1.00	0.12	-	-	-	1.7	1.3	0.8	-	-	-0.6	
49	*cg2462*			TetR-type transcription factor	0	1.12	n.d.	-	-	-	-	-	-	-	-	-	
50	*cg2472*			Predicted hydrolase or acyltransferase	0	1.14	0.48	-	-	-	-	-	-	-	-	-	
51	*cg2576*			DNA polymerase III delta subunit	0	1.17	n.d.	-	-	-	-	-	-	-	-	-	
52	*cg2590*			Putative xanthine/uracil permease	12	1.87	n.d.	-	-	-	-	-	-	0.2	-	-0.6	
53	*cg2591*		*dkgA*	2,5-diketo-D-gluconic acid reductase	0	1.72	n.d.	3.2	0.9	2.0	-	-	-	-	-	-	
54	*cg2674*	ae		Putative Carboxymuconolactone decarboxylase	0	1.08	-0.25	3.0	2.8	2.5	1.5	-	-	-0.9	-	-	McbR*
55	*cg2675*	ae		ABC-type putative peptide transporter, duplicated ATPase component	0	1.70	n.d.	0.8	-0.4	-	3.9	0.8	-	0.2	-	-	McbR*
56	*cg2677*	ae		ABC-type putative peptide transporter, permease subunit	6	2.00	n.d.	-	-	-	3.9	0.8	1.9	2.9	1.6	1.5	McbR*
57	*cg2678*	ae		ABC-type putative peptide transporter, periplasmic subunit	0	1.90	n.d.	1.2	-	-0.2	4.9	1.3	3.4	***6.0***	3.3	2.7	McbR*
58	*cg2687*		*metB*	Cystathionine beta-lyases/cysta-thionine gamma-synthases	0	1.34	0.37	**5.3**	4.2	5.1	-	-	-	-	-	-0.4	McbR*
59	*cg2748*			Hypothetical membrane protein	2	1.21	0.86	-	-	-	-0.4	-	-0.3	2.7	-	1.8	
60	*cg2761*			Metal-dependent hydrolases of the beta-lactamase superfamily III	0	1.05	-0.30	0.4	-0.8	-	-	-	-	-	-	-	
61	*cg2766*			MarA-type transcription factor	0	1.20	0.69	0.7	-	1.5	-	-0.3	-	-	-	-	RamB*
62	*cg2796*	af	*prpD*	Putative 2-methylcitrate dehydratase	0	2.79	n.d.	4.7	-	-	2.0	-	-	-	-	-	DtxR
63	*cg2797*	af		Putative 2-phospho-3-sulpholactate synthase	0	2.92	n.d.	2.4	-	-	-	-0.2	-	-	-	-	DtxR
64	*cg2834*		*cysE*	Serine O-Acetyltransferase	0	1.48	n.d.	-	-0.5	-	3.4	1.3	2.1	0.4	-	-	
65	*cg2835*			Predicted acetyltransferase	0	1.52	0.56	2.5	-	1.9	0.8	-	-	-	-	-	
66	*cg2890*			Putative amino acid processing enzyme	0	2.18	n.d.	-	-	-	-	-	-	-	-	-	
67	*cg2891*		*poxB*	Pyruvate:quinone oxidoreductase	0	2.02	-0.21	3.6	0.4	0.3	0.6	-	0.9	-0.9	-	-	SigB*
68	*cg3049*		*fprA*	putative ferredoxin NADP oxidoreductases	0	1.26	0.24	3.3	3.1	3.6	3.3	3.8	3.0	0.2	2.4	-0.6	
69	*cg3082*	ag		ArsR-type transcription factor	0	3.34	n.d.	-	-	-	-	-	-	-	-	-	DtxR
70	*cg3083*	ag		Predicted Co/Zn/Cd cation transporter	6	3.42	n.d.	-	-	-	1.2	-	-	-	-	-	DtxR
71	*cg3084*	ag		putative FAD dependent NAD(P)H disulphide oxidoreductase	0	3.14	n.d.	-	-	-	2.6	-	-	-	-	-	DtxR
72	*cg3085*	ag		putative FAD dependent oxidoreductase	0	1.47	n.d.	-0.6	-	-	-	-	-	-	-	-	DtxR
73	*cg3112*	ah	*cysZ*	Sulfate permease	7	4.03	0.32	-	-	-	3.8	0.8	2.6	2.6	0.7	1.6	DtxR, McbR*, CysR*
74	*cg3113*	ah		Putative metal chelatase	0	3.31	n.d.	-	-	-	-	-	-	-	-	-	DtxR, McbR*, CysR*
75	*cg3114*	ah	*cysN*	GTPases-Sulfate adenylate transferase subunit 1	0	3.42	0.48	4.4	1.4	3.2	***6.2***	4.1	4.0	***4.6***	3.1	1.0	DtxR, McbR*, CysR*
76	*cg3115*	ah	*cysD*	3-phosphoadenosine 5-phosphosulfate sulfotransferase (PAPS reductase)	0	3.27	0.78	**4.4**	2.8	3.0	***5.5***	4.0	3.7	4.2	2.4	1.0	DtxR, McbR*, CysR*
77	*cg3116*	ah	*cysH*	Phosphoadenosine-Phosphosulfate Reductase	0	2.98	n.d.	-	-0.5	-	2.7	2.3	2.4	-	-	-	DtxR, McbR*, CysR*
78	*cg3117*	ai	*cysX*	Hypothetical protein	0	3.50	1.08	-	-	-	-	-	-	-	-	-	DtxR, McbR*, CysR*
79	*cg3118*	ai	*cysI*	Sulfite reductase hemoprotein beta-component	0	3.50	1.06	0.6	1.5	2.4	3.5	2.1	-0.3	-	-	-	DtxR, McbR, CysR*
80	*cg3119*		*cysJ*	Probable NADPH-dependent Sulfite Reductase	0	2.99	0.29	4.9	4.2	3.5	2.8	1.3	1.4	2.0	1.4	-	DtxR, McbR*, CysR*
81	*cg3157*			Uncharacterized vancomycin resistance protein	1	1.54	0.23	-	-	-	2.7	2.1	3.1	3.7	-0.6	-0.4	
82	*cg3215*		*glpQ1*	Putative glycerophosphoryl diester phosphodiesterase	0	1.02	n.d.	-	-	-	-	-	-	-	-	-	
83	*cg3219*		*ldh*	Anaerobic L-lactate DH	0	1.83	0.12	**5.1**	3.1	**4.8**	4.3	1.3	2.3	-	-	-0.4	GlxR*
84	*cg3227*		*lldD*	Aerobic FMN-L-lactate DH	0	1.14	0.45	1.2	2.1	0.7	***5.2***	4.1	4.0	1.5	-0.6	-	GlxR*
85	*cg3236*		*msrA*	Methionine sulfoxide reductase	0	1.27	1.01	-	-	-0.8	-	-	-	-	-	-	
86	*cg3372*			Hypothetical membrane protein	0	2.80	-0.16	-	-	-	1.8	-	-	-0.7	-	-	McbR*, CysR*
87	*cg3374*	ak		Putative NADH-dependent flavin oxidoreductase	0	2.48	n.d.	-	-	-	-	-	-	-	-	-	McbR*, CysR*
88	*cg3375*	ak		Putative NAD dependent dehydratase	0	1.61	n.d.	1.5	-0.8	0.8	-	-	-	-	-	-	McbR*, CysR*
**Genes repressed at pH 6**																	

1	*cg0148*		*panC*	Pantoate--beta-alanine ligase	0	-1.29	-0.56	-0.6	1.7	-0.8	-	-	-	-	-	-	
2	*cg0244*	a		Hypothetical membrane protein	4	-1.23	-0.49	-	-	-	-	-	-	-	-	-	
3	*cg0245*	a		Putative Moco sulfurase involved in sulphur metal clusters formation	0	-1.02	-0.64	-	-	-	-	-	-	-	-	-	
4	*cg0252*			Hypothetical membrane protein	5	-1.16	-0.58	-	-	-	-	-	-	-	-	-	
5	*cg0308*			Putative membrane protein	4	-1.34	n.d.	-	-	-	-	-	-	-	-	-	
6	*cg0337*		*whiB4*	Transcriptional regulator	0	-1.04	0.39	-	-	-	-	-	-	-	-	-	
7	*cg0350*		*glxR*	Transcriptional regulator	0	-1.18	-0.07	4.0	4.7	4.6	3.3	2.8	3.1	-0.7	-0.6	1.5	
8	*cg0445*	c	*sdhCD*	Succinate dehydrogenase CD	5	-1.31	1.31	-	-	-	1.8	1.4	2.4	3.2	4.0	5.0	RipA, DtxR, RamA*, RamB*
9	*cg0446*	c	*sdhA*	Succinate dehydrogenase A	0	-1.08	1.47	1.0	2.7	1.7	6.7	7.0	***9.0***	***8.5***	9.0	***9.9***	RipA, DtxR, RamA*, RamB*
10	*cg0447*	c	*sdhB*	Succinate dehydrogenase B	0	-1.43	1.14	-	-0.4	-	4.6	5.0	***6.7***	3.5	3.4	5.3	RipA, DtxR, RamA*, RamB*
11	*cg0465*			Conserved hypothetical membrane protein	3	-1.14	n.d.	-	-	-	-	-0.2	-	-	-	-	DtxR
12	*cg0466*			Heme transport system, substrate binding subunit	0	-1.33	n.d.	-	-	-	-	-	-	-	-	-	DtxR
13	*cg0470*	d		Heme transport associated protein	2	-1.66	n.d.	-0.6	1.9	-	3.5	3.8	-	3.3	3.6	-	DtxR, LexA
14	*cg0471*	d		Heme transport associated protein	1	-1.30	n.d.	-	-	-	-	-0.3	-	0.1	1.0	-	DtxR, LexA
15	*cg0493*			Hypothetical protein	0	-1.05	-0.41	-	-	-	-	-	-	-	-	-	
16	*cg0563*	e	*rplK*	50S ribosomal protein L11	0	-1.28	-0.59	-	-	-	0.9	0.7	0.8	-0.9	-	-0.6	
17	*cg0564*	e	*rplA*	50S ribosomal protein L1	0	-1.10	-0.57	4.5	5.0	4.8	3.9	4.2	4.0	4.1	4.4	4.5	
18	*cg0572*	f	*rplJ*	50S ribosomal protein L10	0	-1.60	-0.69	3.4	4.3	3.6	2.1	2.3	2.1	4.2	4.5	3.9	
19	*cg0573*	f	*rplL*	50S ribosomal protein L7/L12	0	-1.53	-0.59	5.3	5.5	4.5	-	1.3	0.0	0.2	0.4	1.6	
20	*cg0582*		*rpsG*	30S ribosomal protein S7	0	-1.07	-0.35	3.1	3.4	2.1	2.2	2.6	3.3	1.3	1.6	1.5	
21	*cg0599*		*rpsS*	30S ribosomal protein S19	0	-1.09	-0.51	1.7	2.2	1.1	-	-	-0.3	-	-	-	
22	*cg0601*	g	*rpsC*	30S ribosomal protein S3	0	-1.05	-0.35	3.3	3.2	3.0	4.7	4.0	4.5	0.3	0.4	0.4	
23	*cg0602*	g	*rplP*	Ribosomal protein L16/L10E	0	-1.02	-0.27	3.1	3.3	3.1	4.5	4.8	5.0	3.0	3.4	3.5	
24	*cg0603*	g	*rpmC*	50S ribosomal protein L29	0	-1.01	-0.20	-	-0.8	-	-	-	-	-	-	-	
25	*cg0604*	g	*rpsQ*	30S ribosomal protein S17	0	-1.18	-0.21	3.3	3.2	2.8	-	-	0.0	-	-	-	
26	*cg0623*	h		ABC-type cobalt exporter unit	8	-1.05	-1.35	-	-	-	-	-	-	-	-	-	
27	*cg0624*	h		Hypothetical membrane protein	2	-1.09	-1.09	-	-	-	-	-	-	1.3	1.6	-0.4	
28	*cg0625*	h		Putative terpene cylase or prenyltransferase subunit	1	-1.28	-1.04	-	2.2	0.0	2.8	3.6	2.2	-	-0.6	-0.4	
29	*cg0690*	i	*groS*	10 kDa chaperonin	0	-1.77	-0.02	4.1	4.3	4.7	-	-	-	-	-	-	SigM*
30	*cg0691*	i	*groEL*	60 kDa chaperonin HSP60	0	-2.19	-0.11	4.0	3.4	3.8	-	-	-	1.2	-0.6	1.1	SigM*
31	*cg0693*	i	*groL1*	60 kDa chaperonin1 Hsp60	0	-1.47	0.02	5.6	5.8	5.8	1.8	0.7	1.8	2.5	1.7	1.6	
32	*cg0748*			ABC-type Fe3+-siderophores transport systems, periplasmic components	0	-1.07	-2.14	-0.3	1.3	-	1.2	0.7	-	0.1	-0.6	-	DtxR
33	*cg0752*			Putative flotillin like protein	1	-1.55	-0.47	0.6	2.7	1.6	3.4	2.3	1.8	4.4	4.7	3.4	
34	*cg0760*		*prpB2*	Methylisocitrate lyase 2	0	-1.54	-0.80	-	3.2	0.9	-0.4	0.8	-	-	-	-	
35	*cg0762*		*prpC2*	2-methylcitrate synthase 2	0	-1.39	n.d.	-	1.7	0.8	-	-	-	-	-	-	
36	*cg0832*			ABC-type transporter, permease components	5	-1.33	-0.40	-	-	-	-0.4	-	-	-	1.0	1.1	
37	*cg0834*			ABC-type transporter, periplasmic component	0	-2.46	-0.03	1.6	3.4	2.7	2.5	3.6	3.7	2.9	4.2	4.1	LexA
38	*cg0842*			Putative DNA helicase	0	-1.03	n.d.	-	-	-	-	-	-	-	-	-	
39	*cg0898*		*pdxS*	Pyridoxal biosynthesis lyase pdxS	0	-1.09	-0.61	4.6	5.0	4.3	-	-	-	-0.7	-	-	LexA, PdxR
40	*cg0952*	o	*mctB*	putative monocarboxylate transporter subunit	2	-2.55	-1.32	-	0.5	-	**2.4**	4.7	4.1	-	2.1	-0.6	
41	*cg0953*	o	*mctC*	monocarboxylate transporter	13	-2.44	-1.01	-	3.0	-0.2	**1.9**	4.7	4.7	3.2	4.5	3.2	
42	*cg0961*			Homoserine acetyltransferase	0	-1.70	n.d.	-	-	-	-	-	-	-	-	-	
43	*cg1072*		*rplY*	50S ribosomal protein L25	0	-1.26	-0.49	0.7	1.1	0.6	-	-	-	-	-	-0.4	
44	*cg1108*		*porC*	Putative secreted protein	0	-1.11	0.06	-	-	-	0.8	2.3	-	-	1.0	-	
45	*cg1122*			Hypothetical protein	0	-1.31	0.23	-	-0.8	-0.8	***3.3***	4.7	***6.1***	5.8	6.3	6.6	
46	*cg1123*		*greA*	Transcription elongation factor	0	-1.21	-0.14	2.0	0.4	2.4	-	-	-	-	-	-	
47	*cg1167*		*metS*	putative methionine transporter subunit	0	-1.04	-1.43	-	-	-	-	-	-	-0.9	-	-0.6	
48	*cg1362*	t	*atpB*	F0F1-type ATP synthase a subunit	6	-1.35	-0.85	-	-	-	2.7	3.2	3.8	5.8	6.0	5.5	SigH*
49	*cg1363*	t	*atpE*	F0F1-type ATP synthase c subunit	2	-1.60	-0.88	-	-	-	4.4	5.0	3.6	***5.4***	6.6	6.4	SigH*
50	*cg1364*	t	*atpF*	F0F1-type ATP synthase b subunit	1	-1.52	-0.96	-0.6	0.5	-0.8	4.5	4.8	4.9	4.9	5.4	**4.3**	SigH*
51	*cg1365*	t	*atpH*	F0F1-type ATP synthase delta subunit	0	-1.26	-1.03	1.9	2.3	1.3	3.9	4.8	4.7	3.2	4.1	3.4	SigH*
52	*cg1366*	t	*atpA*	F0F1-type ATP synthase alpha subunit	0	-1.65	-0.89	4.9	5.1	5.1	**5.6**	6.2	6.1	6.8	6.9	6.4	SigH*
53	*cg1367*	t	*atpG*	F0F1-type ATP synthase gamma chain	0	-1.71	-1.11	1.1	4.2	3.4	***3.3***	4.9	4.7	4.0	4.0	2.5	SigH*
54	*cg1368*	t	*atpD*	F0F1-type ATP synthase beta chain	0	-1.72	-0.94	5.3	5.5	5.4	5.7	5.8	5.6	6.3	6.0	5.7	SigH*
55	*cg1369*	t	*atpC*	F0F1-type ATP synthase epsilon chain	0	-1.09	n.d.	1.6	0.4	0.7	-	-	-0.1	3.1	2.5	1.9	SigH*
56	*cg1436*		*ilvN*	Acetolactate synthase, subunit	0	-1.41	-0.69	2.0	2.2	2.4	1.5	3.2	1.5	-0.9	-0.6	-0.6	
57	*cg1437*		*ilvC*	Ketol-acid reductoisomerase	0	-1.44	-0.91	4.0	3.8	3.9	2.9	3.9	3.3	-0.9	1.7	-0.6	
58	*cg1564*	u	*rpmI*	50S ribosomal protein L35	0	-1.04	-0.31	-	-0.8	-	1.6	1.4	2.4	-	-	-	
59	*cg1565*	u	*rplT*	50S ribosomal protein L20	0	-1.26	-0.29	1.2	1.5	-0.2	-	-0.2	-	-	-	-	
60	*cg1579*			Hypothetical protein	0	-1.36	-0.66	-	-	-	-	-	-	-	-	-	
61	*cg1612*			Putative acetyltransferases	0	-1.32	n.d.	-	-	-	-	-	-	-	-	-	
62	*cg1905*	y		Putative protein kinase	0	-1.33	0.72	-	-	-	-	0.7	-	-	-	-	
63	*cg1906*	y		Putative protein phosphatase	0	-1.17	0.63	-	-	-	-	-0.2	-	-	-	-	
64	*cg1913*			Hypothetical protein	0	-1.19	n.d.	-	-	-	-	-	-	-	-	-	
65	*cg1930*	z		Putative secreted serine protease	0	-1.18	-3.26	-	-	-	-	-	-	-	-	-	DtxR
66	*cg2136*	aa	*gluA*	ABC-type glutamate transporter, ATPase component	0	-1.36	-0.07	-0.6	-0.5	0.8	**1.9**	4.3	3.8	2.2	3.4	2.7	GlxR*, AmtR
67	*cg2137*	aa	*gluB*	ABC-type glutamate transporter, substrate binding component	0	-1.38	-0.39	-0.6	2.7	1.3	2.4	3.3	3.5	4.9	5.5	5.1	GlxR*, AmtR
68	*cg2138*	aa	*gluC*	ABC-type glutamate transporter, permease component	6	-1.19	-0.33	-	-	-	1.8	1.3	3.0	-	-	-0.6	GlxR*, AmtR
69	*cg2167*		*rpsO*	30S ribosomal protein S15	0	-1.01	-0.14	1.1	1.8	1.9	-0.4	-	-	-	-0.6	0.6	
70	*cg2181*			ABC-type peptide transporter, periplasmic component	0	-2.20	-0.53	-0.2	3.5	1.5	2.8	4.0	4.1	***3.9***	5.7	5.6	AmtR
71	*cg2234*			ABC-type cobalamin/Fe3+-siderophores transporter, secreted component	0	-1.32	n.d.	-0.6	-	-	-0.4	0.7	-	-0.9	-0.6	-	DtxR, RamB*
72	*cg2235*		*rplS*	50S ribosomal protein L19	0	-1.40	-0.46	2.5	2.2	1.9	6.6	6.9	7.5	3.0	3.9	3.7	
73	*cg2253*		*rpsP*	30S ribosomal protein S16	0	-1.14	-0.34	4.1	4.7	4.0	1.2	1.3	2.5	1.6	-0.6	-0.4	
74	*cg2467*	ac		ABC-type peptide transporter, substrate binding component	0	-1.21	-0.17	-	-	-	-0.4	2.8	2.8	1.4	-0.6	2.1	
75	*cg2470*	ac		ABC-type peptide transporter, substrate binding component	0	-1.66	-0.36	0.7	2.2	1.2	1.2	1.3	0.0	2.8	3.4	3.4	
76	*cg2559*	ad	*aceB*	Malate synthase G	0	-1.21	n.d.	-0.6	4.7	2.0	-	-	-	-	-	-	RamA, RamB
77	*cg2560*	ad	*aceA*	Isocitrate lyase	0	-2.73	-2.19	-	4.9	2.4	-	-0.3	-	-	-	-	RamA, RamB
78	*cg2573*		*rpsT*	30S ribosomal protein S20	0	-1.33	-0.56	2.8	3.3	2.1	2.7	2.7	3.5	1.6	1.4	1.2	
79	*cg2603*		*ndk*	Nucleoside diphosphate kinase	0	-1.19	-0.20	2.8	3.3	3.2	-	-	-	-	-	-	
80	*cg2647*		*tig*	Trigger factor	0	-1.47	-0.27	4.8	5.4	5.0	-	-	-	-	-	-	
81	*cg2703*			ABC-type transporter, permease component	6	-1.90	-0.32	-	-	-	-	-	-	-	-	-	
82	*cg2705*			ABC-type transporter, periplasmic component	0	-1.68	-0.28	**4.9**	7.0	**5.8**	***6.9***	7.7	8.0	**6.8**	7.6	7.4	
83	*cg2840*		*actA*	Butyryl-CaA-acetate coenzyme A transferase	0	-1.67	-0.01	***6.4***	7.1	***7.6***	**3.4**	4.9	**3.2**	-	2.0	0.4	RamB*
84	*cg2953*		*xylC*	Benzaldehyde dehydrogenase	0	-1.39	0.61	1.6	3.3	4.1	1.3	4.3	3.7	-	-	1.5	GlxR*
85	*cg3011*		*groL2*	60 kDa chaperonin 2 (HSP60)	0	-2.20	-0.44	***5.9***	6.7	6.8	-0.4	1.7	3.5	2.2	2.4	3.0	
86	*cg3048*		*pta*	Phosphate acetyltransferase	0	-1.07	0.16	-	1.7	2.3	-	2.9	2.8	-	-	-	RipA, RamA, RamB
87	*cg3096*			NAD-dependent aldehyde dehydrogenases	0	-3.24	0.10	***3.2***	6.6	5.9	***3.0***	6.1	5.7	-	1.0	1.4	
88	*cg3107*		*adhA*	Zn-dependent alcohol dehydrogenases	0	-1.97	0.06	-	2.9	1.8	-	5.6	4.9	-	3.7	-0.4	RamA, RamB, GlxR*
89	*cg3156*		*htaA*	secreted protein implicated in iron acquisition and transport	0	-2.50	-2.76	-	-	-	-	-	-0.3	**2.7**	5.7	-	DtxR
90	*cg3195*			Flavin-containing monooxygenase	0	-2.23	0.32	-	2.2	-0.8	-	4.9	3.9	-	3.7	2.1	
91	*cg3212*			Hypothetical membrane protein	0	-1.92	-0.50	-	-	-	-	-	-	-	-	-	

Genes for which an increased or decreased mRNA level was found at pH 6 in comparison to pH 7.5. The gene locus tag, organisation in operons, the gene name, the (proposed) function of the protein as well as the predicted number of transmembrane helices are given. The results of the transcriptome analysis are given as induction factor at pH 6 and pH 9 in comparison to pH 7.5. Results of the proteome analysis are indicated for the soluble, membrane and envelope fraction. Regulators of particular genes are given based on the CoryneRegNet data base. For further details see legend of Table 1.

In addition, a number of proteins with changed abundance was detected for which no change in gene transcription was observed. We identified for 43 proteins increased and for 30 proteins decreased peptide numbers at pH 6 (Additional file 1). The same held for 32 proteins with increased and for 20 proteins with decreased peptide numbers under alkaline conditions, (Additional file 2). An example is the gene *cg1111 *encoding enolase. The mRNA content was neither significantly changed at pH 6 (m-value 0.24) nor at pH 9 (m-value 0.09) but 229 peptides were found in the cytoplasmic fraction at pH 7.5, 334 at pH 6, and 104 at pH 9 (Additional file 2). Other examples with stable mRNA level and varying peptide numbers include the porines of the outer membrane PorA and PorH (decreased amounts of peptides found at pH 9 and 6 in comparison to pH 7.5 in the membrane fraction) as well as MetE (increased peptide numbers at pH 6 in the cytoplasmic fraction, Additional file 2). This indicates that posttranscriptional or posttranslational control might be involved and that the regulation of protein stability is important during pH acclimatization. Furthermore, because of the (putative) function of many genes that are differentially expressed in a pH dependent manner the rearrangement of the cell wall might take place and influence the gene expression response.

Subsequently, we checked whether transcriptional regulators are known to be involved in expression control of genes that were found to be regulated. This was done using the CoryneRegNet data base which provides information on 72 regulators in *C. glutamicum *[18]. For 21 of the 39 genes found to be repressed at pH 9, predictions were made or experimental evidence was obtained, for regulation by particular transcription factors (Table 1). Accordingly, for approx. 50% of the genes found to be induced at pH 9 or differentially expressed at pH 6 the transcriptional regulator was proposed or identified (Table 1, 2).

### Iron homeostasis of *C. glutamicum *is affected by the external pH

The iron availability is monitored in *C. glutamicum *by the binding of ferrous iron to the transcription factor DtxR [19]. At high internal concentrations of ferrous iron, the regulator binds to operator sites in the promoter regions of target genes, including RipA, the second regulator of iron homeostasis. Whereas DtxR can act both as repressor and activator, RipA acts as repressor only [20,21]. The combined transcriptome and proteome data suggest that the external pH value influences the availability of iron. At alkaline pH, DtxR-repressed genes like *cg0925-28*, encoding a siderophore ABC transporter, or *cg0767*, encoding a siderophore interacting protein, are found to be repressed, while the mRNA levels respectively peptide numbers of DtxR-activated genes like *ftn *(encoding a ferritin-like protein involved in iron storage) and *dps *(*cg3327*) are increased. We found RipA-regulated genes encoding iron containing enzymes like succinate dehydrogenase (*cg0446-0447*), aconitase (*cg1737*), or catalase (*cg0310*) to be (slightly) repressed at pH 6, whereas the same genes were found to be induced at alkaline pH (Table 1). Additionally, higher peptide numbers were found for SdhA, SdhB, Acn, and KatA under alkaline conditions (Table 1). Furthermore, genes of the SufR regulon, *cg1759-65*, including the genes *nifS2*, *sufC*, and *sufB *which encode components of the FeS cluster assembly machinery, as well as the regulator SufR itself are induced at pH 9 (Table 1). In contrast, the ABC type transporter for ferric iron uptake encoded by *cg0508-0506 *is not under the control of DtxR and no change of the transcript or protein level was detected (data not shown). In summary, we found a pH-dependent regulation of genes of the RipA and DtxR regulon indicating the activation of the iron starvation response at pH 6 and iron excess conditions at pH 9.

### At neutral and acidic pH H_2_O_2 _can be detected in *C. glutamicum *cultures

The induction of iron starvation response at pH 6 was surprising because the solubility of iron is increased at low pH values and the availability should be increased at pH 6. Therefore, we speculated that activation of iron starvation could be caused by an impaired function of the cytoplasmic regulators. By oxidation of the cytoplasmic ferrous iron to ferric iron, the co-activator of DtxR, DtxR-mediated regulation might be triggered. Such a process could be induced by the endogenous formation of reactive oxygen species as described for the Fur protein in *E. coli *[22]. In order to test for the pH dependent occurrence of oxidative stress in *C. glutamicum *cells, we performed again batch fermentations in bioreactors in minimal medium under continuous pH control. During the exponential phase we detected significantly higher levels of H_2_O_2 _at pH 6 (6.5 μM, OD_600 _4) than under neutral (2.2 μM, OD_600 _12) or alkaline pH conditions (0.9 μM, OD_600 _6). Additionally, in cultures grown in buffered minimal medium in Erlenmeyer flasks we could detect H_2_O_2 _during exponential growth in *C. glutamicum *cultures. We measured 3 μM H_2_O_2 _in cultures grown at pH 9 but in cultures grown at pH 7.5 and pH 6 we measured unexpected high concentrations of H_2_O_2_, namely 20 μM after eight hours of incubation. The results indicate the increased occurrence of oxidative stress in *C. glutamicum *and/or suggest that the defense against oxidative stress is impaired in a pH dependent manner. In order to assess an effect of H_2_O_2 _production at low pH we applied a well established method for the measurement of protein carbonylation by using the OxyBlot assay. Total proteins of cells grown at pH 6, 7.5 and 9 were extracted and subjected to 1D SDS PAGE before and after the OxyBlot treatment (Additional file 3). Interestingly, a high number of proteins can be detected to harbour carbonyl groups in *C. glutamicum *protein extracts of cells grown at every pH. We could not find a significant increase in carbonylation at low pH.

**Figure 3:**
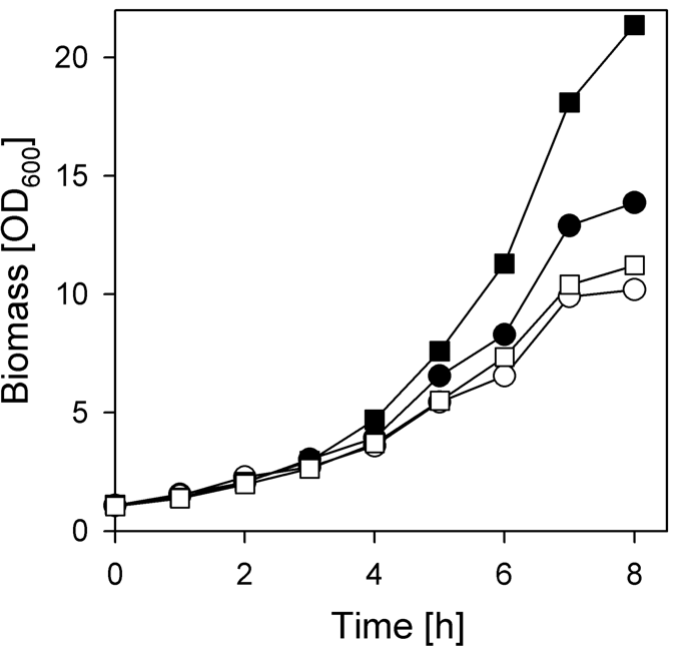
**Impact of externally added catalase enzyme on growth of *C. glutamicum***. Wild type cells were exposed to pH 6.0 (white symbols) and 7.5 (black symbols) in buffered medium in Erlenmeyer flasks and growth was determined in absence (circles) or presence (squares) of purified catalase protein of *C. glutamicum*. The enzyme (16 KU/ml) was added at the beginning.

Furthermore we performed growth experiments in Erlenmeyer flasks at pH 7.5 and pH 6 in presence of external catalase enzyme (Fig. 3). Interestingly, for *C. glutamicum *cells grown at pH 7.5 in presence of catalase (16 KU/ml) a higher growth rate was observed (μ = 0.393 ± 0.005) in comparison to the absence of external catalase (μ = 0.343 ± 0.006). At pH 6 addition of catalase had no significant beneficial effect because the growth rates in presence or absence of catalase were comparable (Fig. 3). Catalase was also added after every hour of incubation in order to prevent loss of enzymatic activity and to provide continuous catalase activity but comparable results were obtained (data not shown). In conclusion, elimination of H_2_O_2 _by addition of external catalase enzyme facilitates growth of *C. glutamicum *at neutral pH but not at acidic pH conditions.

### Metabolic alterations during response to acidic pH

The amounts of several enzymes were found to be affected by the changed external pH including succinate dehydrogenase and aconitase. In order to unravel metabolic alterations caused by the differing protein content, we performed GC-MS based metabolic profiling of cells grown at pH 6 and pH 7.5 under continuous pH control. Thereby, we identified numerous amino acids, intermediates of TCA, glycolysis, pentose phosphate pathway, and methionine pathway to be present at significantly different levels (Table 3). For example, pyruvate was found at pH 6 at an eleven fold higher concentration than at pH 7.5. Within the TCA, citrate, which is the substrate of aconitase, was found to accumulate like malate and fumarate. In contrast the metabolites 2-oxoglutarate and succinate were found in significantly lower concentrations at pH 6 (Table 3). Among the amino acids accumulation of phenylalanine, valine, glutamine, and alanine was observed, and proline and β-alanine were found in lower concentrations at pH 6. The pool size of methionine was slightly decreased, but we identified intermediates of the methionine pathway to be present in high concentrations like cystathionine and cysteine (Table 3). On the other hand nearly all enzymes of the methionine pathway were found to be induced at the mRNA and/or protein level.

**Table 3:** Differential metabolite pattern at pH 6 in comparison to pH 7.5

Metabolite	differential content	t-test
Methionine synthesis		
homolanthionine	-15.28	0.0392
Serine	-1.84	0.0307
Glycine	-1.67	0.0010
homocysteine	-1.41	*0.2019*
methionine	-1.09	*0.9449*
threonine	1.26	0.0302
Aspartate	1.33	*0.1295*
homoserine	1.58	*0.3969*
O-acetyl-serine	2.34	0.0165
S-adenosyl-homocysteine	2.45	0.0441
cysteine	2.46	0.0030
O-acetyl-homoserine	3.71	0.0002
cystathionine	6.96	0.0044
		
**Glycolysis and Pentosephosphate pathway**		

Pep	-3.58	0.0025
DHAP	-2.89	0.0004
DHAP	-2.79	0.0010
glycerate-3-P	-1.99	0.0043
gluconate-6-P	-1.77	*0.1733*
ribose-5-P	-1.44	0.0373
fructose-1-6-P	2.07	0.0360
glycerate-2-P	2.86	*n*.*d*.
fructose-6-P	3.00	0.0009
glucose-6-P	3.18	0.0000
glucose-6-P	3.85	0.0006
pyruvate	11.10	0.0341
		
**TCA cycle**		

alpha-ketoglutarate	-6.07	0.0131
succinate	-5.37	0.0039
fumarate	1.25	*0.1755*
citrate	1.84	*0.0775*
malate	2.06	0.0065
		
**Amino acids**		

proline	-8.67	0.0006
beta-alanine	-7.30	0.0000
ornithine, citrulline, arginine	-2.38	*0.2871*
serine	-1.84	0.0307
glycine	-1.67	0.0010
lysine	-1.58	*0.3466*
asparagine	-1.45	*0.3712*
methionine	-1.09	*0.9449*
leucine	1.01	*0.9353*
glutamate	1.02	*0.7068*
tyrosine	1.17	*0.1464*
threonine	1.26	0.0302
L-aspartate	1.33	*0.1295*
tryptophan	1.40	*0.0606*
isoleucine	1.73	0.0007
histidine	2.28	*0.2898*
cysteine	2.46	0.0030
alanine	3.28	0.0117
glutamine	4.85	0.0002
valine	5.52	0.0013
phenylalanine	9.08	0.0020

Pool size ratios determined by GC-MS in extracts of *C. glutamicum *cells grown at pH 7.5 and pH 6. The ratios have been calculated using the mean values from two independently grown cultures and three technical replicates each. A t-test was applied for determining if observations were significantly different. Numbers in italics indicate values above the chosen significance cut off (P < 0.05), n.d. means not determined.

### The McbR regulon is induced at acidic pH

**Figure 4:**
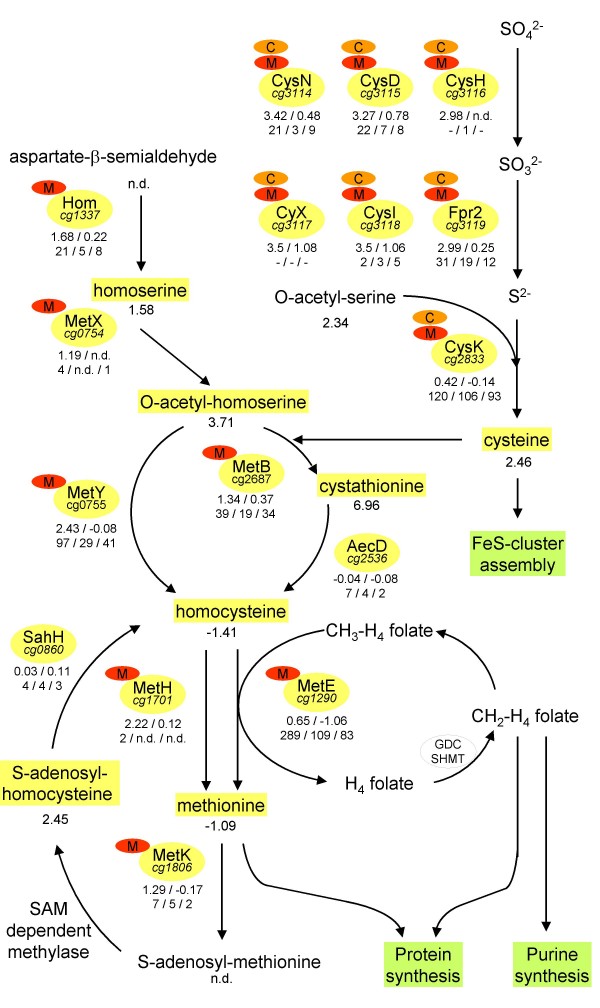
**The pH dependent regulation of the methionine and cysteine metabolism in *C. glutamicum***. The metabolite pool sizes at pH 6 in comparison to pH 7.5 are indicated below the intermediates. The involved proteins as well as the encoding genes are given in circles and the regulation by McbR (M) and/or CysR (C) is indicated. Below the proteins the relative expression levels at pH 6 and pH 9 in comparison to pH 7.5 are given and the peptide numbers detected in the soluble protein fraction at pH 6/7.5/9 are given. (n.d. means not detected)

At pH 6 we observed induction of genes encoding proteins of the methionine and cysteine pathway (Table 2, Fig. 4). Intermediates of these pathways are involved in essential cellular functions including the assembly of iron sulfur clusters (cysteine), the *de novo *synthesis of proteins (cysteine, methionine) or the metabolism of C_1 _units (*S*-adenosyl-methionine, methyltetrahydrofolate; Fig. 4). Many of the genes are under control of McbR and the ancillary regulators CysR and SsuR [23-25]. Among them are, e.g., the *fpr2*-*cysIXHDNYZ *cluster and *cysK*, encoding the sulfate permease CysZ, the complete set of enzymes involved in sulfate reduction to sulfide (CysDN, CysH, CysIX, and Fpr2) as well as the serine-*O*-acetylserine sulfhydrylase CysK, involved in cysteine synthesis (Fig. 4). Furthermore, the genes *hom*, *metB, metH, metK, metXY*, and *metQN*, encoding enzymes of the methionine pathway and subunits of the primary methionine uptake system MetQNI, were found to be induced [8,26]. The genes encoding the cysteine synthase (*cysK*), the homocysteine methyltransferase (*metE*), the β-C-S lyase (*aecD*), and the *S*-adenosyl-homocysteine hydrolase (*sahH*) were not found to be induced at the mRNA level (Table 2, Fig. 4). Corresponding to the unaffected mRNA level of *aecD *and *sahH *no differential peptide numbers were found (Fig. 4). For the AecD enzyme we determined unchanged cystathionine lyase activities in cells grown at pH 7.5 and at pH 6 using an enzymatic assay (data not shown). However, a higher protein level was found for MetE and a lower amount for CysK in spite of the unaffected mRNA levels (Additional file 1 and 2, Fig. 4). This might be an indication for increased protein stability of MetE and CysK at low pH. In contrast to pH 6 the McbR and CysR regulon were not found to be differentially expressed at pH 9 (Table 2, Fig. 4). It should be noted that we are not able to report on genes under the control of the transcription factor SsuR, because no transcription data were obtained for these genes and no peptides were found representing the corresponding proteins.

At the metabolite level we observed the accumulation of intermediates of the methionine pathway upstream of the AecD enzyme including L-homoserine, O-acetyl-L-homoserine, L-cysteine, and L,L-cystathionine (Table 3, Fig. 4). Furthermore, the content of the McbR effector S-adenosyl-homocysteine was increased at low pH. In contrast, the pool sizes of homocysteine and methionine, representing metabolites downstream of AecD, were found to be slightly reduced.

**Figure 5:**
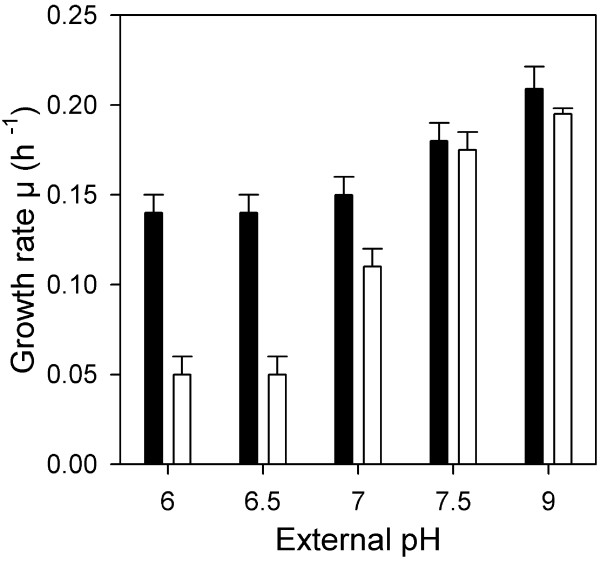
**The pH dependent impact of cysteine on growth of *C. glutamicum***. Wild type cells were exposed to different pH values in MTP and growth rates were determined in absence (black bars) or presence 10 mM cysteine (white bars).

From the observed metabolic imbalance we inferred that accumulation of intermediates of the methionine pathway upstream of AecD or the lower pool size of the downstream intermediates could contribute to the growth defect of *C. glutamicum *cells at acidic pH. In order to test this hypothesis we performed growth experiments at pH 7.5 and 6 in absence or presence of 10 mM cystathionine, cysteine, homocysteine, or methionine. Based on these assumptions, the addition of cystathionine or cysteine should increase pH dependent growth inhibition whereas homocysteine and methionine should supplement a putative demand for these compounds at pH 6. The addition of cystathionine, homocysteine and methionine had no significant effect on *C. glutamicum *growth at pH 6 (data not shown). However, addition of cysteine significantly decreased growth rates of cells exposed to acidic pH values. Further experiments revealed that the extent of growth inhibition by cysteine was indeed pH dependent. Whereas at pH 9 and 7.5 cysteine addition had no effect on the growth rate, at pH 7 growth was retarded and at pH 6.5 and 6 cells were hardly able to grow (Fig. 5).

### Differential expression of further regulatory modules

Beside the induction of methionine and cysteine synthesis, the complete *arg *cluster was found to be induced at pH 6. The expression of the *arg *genes, encoding all enzymes for synthesis of arginine from glutamate via the urea cycle, was proven to be under the control of the two repressors ArgR and FarR (Table 2). The investigation of the metabolite pattern revealed, however, a lower pool size for ornithine, citrulline, and/or arginine, represented by only one signal in the GC-MS analysis (Table 3).

The transcription factors RamA and RamB as well as GlxR are major regulators of the carbon flux in *C. glutamicum *[19,27,28]. At pH 9 and pH 6 we observed the repression of several genes indicating alterations of the carbon metabolism. Among them are *aceA*, encoding isocitrate lyase, *aceB*, encoding malate synthase, and *mctC*, encoding an uptake system for pyruvate, acetate and propionate [8,29]. All of these genes are under the control of RamA and RamB. Additionally, alternative oxidases were found to be induced, like the FMN containing lactate dehydrogenase LldD and the pyruvate quinone oxidase Pqo. Whereas *lldD *expression was proposed to be under the control of GlxR, *pqo *expression was found to be part of the sigma factor SigB regulon [28,30].

Another regulatory module found to be induced at pH 6 comprises the genes *cg1214-18*. These genes encode the NadAC proteins, involved in NAD synthesis, a putative cysteine desulfurase, possibly involved in maturation of FeS clusters necessary for function of the NadAC complex, and the regulator NrtR [8,31]. All genes of the operon were induced at pH 6 and for NadC (*cg1215*), an increased number of peptides (33) was found at acidic conditions in comparison to pH 9 (12 peptides, Table 1). Under alkaline conditions genes of the NrtR regulon were not induced. Subsequently, we determined the cellular concentration of all NAD derivatives in *C. glutamicum *cells grown at pH 7 and pH 6. Whereas the NADP and NADPH concentrations at pH 6 were only half of those observed at pH 7 (NADP pH 6: 0.11 ± 0.01 mM, pH 7: 0.18 ± 0.03 mM; NADPH pH 6: 0.25 ± 0.03 mM, pH 7 0.48 ± 0.07 mM) the NAD and NADH concentrations were only one third at pH 6 in comparison to pH 7 (NAD pH 6: 0.57 ± 0.05 mM, pH 7: 1.53 ± 0.14 mM; NADH pH 6: 0.46 ± 0.05 mM, pH 7 1.49 ± 0.39 mM). Calculation of the ratios of the oxidized and reduced forms revealed that the reduction state of the cell was not affected by acidic pH. However, significantly lower levels of NAD derivatives were found under acidic conditions accompanied by the induction of genes encoding enzymes involved in the first steps of their synthesis.

## Discussion

The purpose of this study was to achieve a general conception of the acclimatization of the Gram-positive soil bacterium *C. glutamicum *towards acidic as well as alkaline external pH on the transcriptome, proteome, as well as on the physiological level. Of particular interest was the question which metabolic processes are impaired under conditions of non-optimal pH and thereby represent limitations for growth. As a prerequisite the growth optimum was determined and found to be in the range between pH 7 and 8.5. Consequently, *C. glutamicum *can be regarded as a moderately alkali-tolerant strain in comparison to *E. coli *with a pH optimum at 6-7 [1]. In *E. coli *the capacity of pH homeostasis is higher than in *C. glutamicum*, because *E. coli *can maintain pH_i _values of 7.6 ± 0.2 at external pH values ranging from pH 5 to 9 [32]. Consequently, mechanisms of pH homeostasis are less effective in *C. glutamicum *in comparison to *E. coli*. In agreement with the observed growth optimum, pH homeostasis was effective in a range between 6 and 9 in *C. glutamicum*. At lower or higher external pH values maintenance of the internal pH at a level of 7.5 was not achieved leading to reduced growth rates. In order to identify targets which are affected by pH values exceeding the boundaries of effective pH homeostasis in *C. glutamicum*, we checked at first whether the proton motive force (pmf) was affected in a pH-dependent manner. The pmf is the major driving force for the generation of ATP by oxidative phosphorylation. However, the pmf was kept constant over a surprisingly broad pH range.

Subsequently, the dissection of pH acclimatization by transcriptome and proteome studies uncovered many physiological processes that are affected in a pH-dependent manner. The gene expression pattern observed in this work overlaps with the results obtained for gene expression analysis during growth on lactate at pH 5.7 [11]. Beside a high number of genes which seem to be expressed in dependence of the carbon source, 15 out of 88 genes which were found in our studies to be transcriptionally induced at pH 6 were also identified during growth on lactate as induced at low pH [11]. On the other hand, 31 out of 91 genes that were found to be repressed under acidic conditions were also found to be repressed by Jakob *et al*. (2007). Among them are genes encoding subunits of the succinate dehydrogenase, the F_1_F_0_-ATPase, and rRNA genes. For several of them the expression was shown to be dependent on the growth rate which is in agreement with a lower expression at pH 6 and pH 9 at which we observed lower growth rates than at neutral pH [10]. The evaluation of differential transcript and protein patterns by the comparison with targets of transcriptional regulators in *C. glutamicum *unraveled numerous regulatory modules that are activated during pH acclimatization. Novel findings are represented by the induction of the iron starvation response as well as the induction of expression of the methionine and cysteine pathway under acidic conditions.

### At neutral and low pH *C. glutamicum *is impaired by oxidative stress

The permanent formation of H_2_O_2 _in living cells was already previously described for *E. coli*. However, the detection of hydrogen peroxide in the medium was only possible after inactivation of the primary H_2_O_2 _scavenging enzyme alkyl hydroperoxide reductase (AhpCF) [33]. Homologues of the Ahp proteins are missing in *C. glutamicum *and we found that WT cells produce significant amounts of H_2_O_2 _at neutral and especially at acidic pH. The formation of H_2_O_2 _can cause cellular damage by oxidation of sulfur atoms in cysteine or methionine residues at the protein surface, by protein carbonylation, or by oxidation of iron sulfur clusters [34]. The analysis of protein carbonylation was performed for the first time for *C. glutamicum *and in contrast to other bacteria a high number of carbonylations were detected under all conditions [35]. In consequence, *C. glutamicum *might be exposed towards a certain level of oxidative stress under all our experimental conditions and the pH dependent differences might be overlooked.

The most likely source for the formation of hydrogen peroxide is not the respiratory chain but alternative oxidases like the lactate oxidase and the pyruvate oxidase. Both enzymes are utilized by lactic acid bacteria in order to excrete large amounts of H_2_O_2 _[36,37]. In *C. glutamicum *lactate oxidase as well as pyruvate oxidase were found to be significantly induced at low pH. The decrease of the internal pH in *C. glutamicum *cells at an external pH of 6 might also cause formation of reactive oxygen species by soluble oxidoreductases, especially those using FADH_2 _as cofactor, upon malfunctions at non optimal pH conditions [34,38]. Furthermore, the catalase content of cells is reduced at pH 6 in comparison to pH 7.5 and even more to pH 9. Thereby, an additional decrease of the H_2_O_2 _scavenging capacity at neutral and low pH is expected. Accordingly, addition of catalase enzyme at pH 7.5 caused a significant increase in growth rate pointing to the limitation of growth by H_2_O_2 _formation under neutral conditions and the insufficient activity of the cellular catalase enzyme. At pH 6 production of H_2_O_2 _is accompanied by other limitations for *C. glutamicum *cells and consequently, addition of catalase did not increase growth rate significantly.

**Figure 6:**
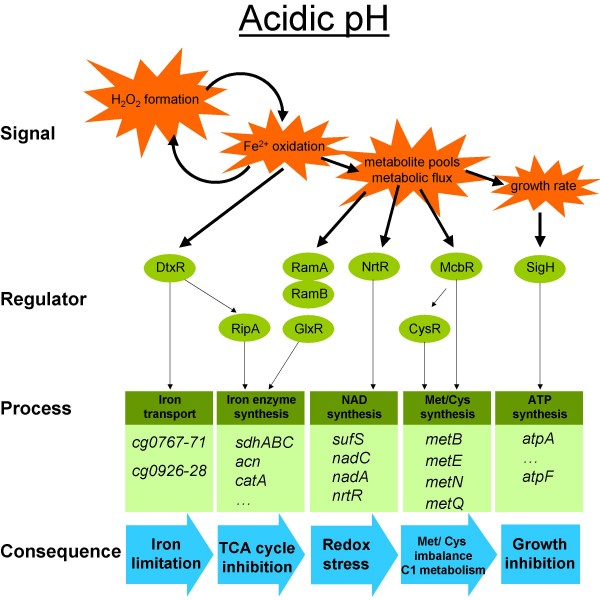
**Regulatory modules activated under acidic conditions in *C. glutamicum***. Putative stimuli and signals are indicated as stars, regulators involved are shown in circles. The affected processes and genes are indicated in boxes and the physiological consequences are given at the bottom.

Additional limitations at pH 6 could be cause by the disruption of iron sulfur clusters by ROS or by oxidation of the amino acids cysteine and methionine. In agreement with this, expression of the methionine sulfoxide reductase which is involved in repair functions is induced at low pH (Table 1). Thioredoxins are in general thought to be involved in this process but were also found to be not induced by H_2_O_2 _in other bacteria like in *Bacillus subtilis *[39]. Additionally, NAD synthesis in *C. glutamicum *potentially depends on iron sulfur clusters [40] and these seem to be affected by H_2_O_2_. The total NAD concentration was found to be reduced significantly. In agreement to this, in *E. coli *the NadA enzyme was identified as a target of oxidative stress [41]. The formation of H_2_O_2 _in *C. glutamicum *cells might impair the function of the enzymes NadC and/or NadA directly or indirectly because significantly reduced levels of NAD derivatives were found. Consequently, induction of the NrtR regulon was found (Fig. 6).

### The occurrence of oxidative stress interferes with iron availability and control of metabolic fluxes in *C. glutamicum*

We propose that the link between acidic pH and iron starvation response may be caused be the H_2_O_2 _dependent conversion of ferrous into ferric iron, resulting in the inactivation of DtxR and the consecutive activation of RipA, the two regulators of iron homeostasis in *C. glutamicum *(Fig. 6). In agreement with our assumption is the observation that the addition of higher amounts of iron sulfate at low pH values did not diminish the expression of DtxR controlled genes [11]. We observed a reduced transcription of the *acn *and *sdhABC *genes which was correlated with lower contents of the corresponding proteins (Table 2), due to the lack of DtxR-mediated activation and repression by RipA. In turn, this could cause a reduced activity of the TCA cycle and indeed we could determine lower levels of α-ketoglutarate and succinate and drastically increased levels of pyruvate and higher levels of citrate and malate (Fig. 6). As a consequence of the increased pyruvate pool, alternative metabolic routes are activated. Among them is the oxidation of pyruvate by the pyruvate oxidase Pqo or the synthesis of the branched chain amino acids valine and isoleucine at pH 6 (Table 3).

In conclusion, at acidic pH endogenous formation of hydrogen peroxide occurs in *C. glutamicum *cells at an extent that can obviously not be compensated by ROS defense mechanisms. As a consequence the induction of the iron starvation response was observed including lower levels of iron containing enzymes of the TCA cycle, and oxidative damage of iron sulfur cluster containing enzymes as proposed for the NAD synthesis pathway. All effects represent metabolic limitations and contribute to the impaired growth of *C. glutamicum *cells under acidic conditions (Fig. 6).

### Cysteine accumulation inhibits the cysthationine-β-lyase AecD and causes additional limitations for growth of *C. glutamicum *under acidic conditions

High internal concentrations of H_2_O_2 _can cause DNA damage and require DNA repair as well as *de novo *synthesis [42]. Newly synthesized DNA is methylated whereby S-adenosylmethionine (SAM) serves as the methyl donor [43]. We could not measure SAM directly but found that the corresponding pool size of the resulting intermediate S-adenosylhomocysteine (SAH) was increased at acidic pH. This increase causes inactivation of the McbR repressor and led to the transcriptional induction of genes of the methionine and cysteine pathway (Fig. 4) [23].

An exception is the *aecD *gene encoding the cystathionine-β-lyase AecD which is not under McbR control. In agreement with this, the AecD protein content was unaffected by changed pH values and comparable enzyme activities were determined under neutral and acidic pH conditions. The increased pool sizes of metabolites upstream of AecD including cystathionine and cysteine and slightly reduced pool sizes of the downstream metabolites homocysteine and methionine could be caused by the missing induction of the *aecD *gene at acidic pH and/or by inhibition of the AecD enzyme activity. As a consequence, accumulation of cysteine was found, indicating an imbalance of thiol homeostasis in *C. glutamicum *under acidic stress conditions. High levels of cysteine can cause oxidative stress by formation of H_2_O_2 _and hydroxyl radicals via the Fenton reaction which, concomitantly, would increase damage of proteins and DNA [44]. Consequently, cysteine would be converted into cystine. Because cystine is preferred over cystathionine by AecD, this could cause inhibition of methionine and SAM biosynthesis as well as further cysteine accumulation [45]. Internal accumulation of cysteine was found to be toxic for *E. coli *cells [44]. In conclusion, cysteine addition would result in its accumulation in *C. glutamicum *and would thus amplify oxidative stress at acidic pH and thereby cause the severe growth inhibition.

Interestingly, methionine synthesis was also affected at acidic pH in *E. coli *and, in this case, accumulation of homocysteine was observed [14]. This indicates that the homocysteine methyltransferase MetE is affected at low pH. Because the MetE protein represents the major target for oxidative stress in *E. coli *we assume that oxidative stress may occur in this strain at low pH as well [46,47]. In contrast to *E. coli*, in *C. glutamicum *inhibition of the AecD enzyme was found. This suggests that a significant flux from homoserine to homocysteine via trans-sulfuration occurs at least during growth at pH 6. In addition, AecD inhibition prevents the accumulation of homocysteine which is more toxic for bacteria than cystathionine [14,48].

## Conclusions

At non-alkaline pH values oxidative stress was found to occur in *C. glutamicum *and the reactive oxygen species defense was found to be impaired. As a consequence maintenance of cellular NAD levels is impaired and iron starvation response is activated. This leads to reduced protein levels of iron-containing enzymes affecting the TCA cycle and other metabolic pathways at low pH, among them methionine synthesis. McbR-dependent activation leads to cysteine accumulation which is toxic under acidic conditions. We thus have unraveled regulatory modules activated during acidic pH response in *C. glutamicum *(Fig. 6) and have identified targets as well as physiological consequences for the cellular stress response. Beside insights into bacterial physiology conclusions can be drawn for acclimatization of pathogenic Actinomyces and for the optimization of biotechnological production processes.

## Methods

### Strains and culture conditions

Strain ATCC 13032 served as *Corynebacterium glutamicum *wild type. *C. glutamicum *cells were grown either in Brain Heart Infusion (BHI) medium (Becton-Dickenson, Heidelberg, Germany) or in minimal medium MM1 [49] at 30°C. For all experiments *C. glutamicum *cells were precultured in 5 ml BHI medium for approx. 8 h and subsequently used for inoculation of 20 ml MM1. After approx. 20 h the culture was used to inoculate fresh MM1 medium of a desired pH to an OD_600 _of 1-2. Batch cultivations at different pH were performed in 2 l stirred bioreactors (Biostat B, Sartorius, BBI Systems, Melsungen, Germany) under continuous control of pH (6, 7.5, or 9) temperature (30°C) and pO_2 _(>30%) at a flow rate of 1 vvm air. Growth was followed by measuring the optical density at 600 nm (OD_600_). Each cultivation at pH 6, 7.5, and 9 was performed twice and parameters were determined always in triplicate. In order to screen for the impact of amino acid addition on growth at different pH values cultivations were performed in 96 well micro titer plates in a volume of 200 μl MM1 medium and the OD_600 _was followed by using a plate reader. Growth was also investigated in Erlenmeyer flasks (20 ml) MM1 medium and purified *C. glutamicum *catalase enzyme was a kind gift of Roche Diagnostics, Manheim, Germany.

### Determination of bioenergetic parameters

During the exponential phase of growth cells were harvested, washed twice and resuspended in 100 mM MES buffer of the respective pH. Cell volumes were determined by the distribution of ^3^H-labelled H_2_O (0.55 mCi/l) and ^14^C-labelled inulin (0.14 mCi/l) between the cell pellet and the supernatant. The membrane potential was determined by measuring the distribution of ^14^C-labelled TPP (5 μM final concentration, sp. radioactivity 0.995 Ci/mol). Processing of samples for rapid separation of extra- and intracellular fluids was performed by using silicone oil centrifugation with perchloric acid in the bottom layer (Rottenberg, 1979). Internal pH was determined by measuring the distribution of ^14^C-labelled benzoic acid (15 μM final concentration, sp. radioactivity 3.12 Ci/mol). All measurements were performed at least in triplicate and standard deviations were calculated.

### Detection and elimination of H_2_O_2_, protein carbonylation and cysthationine lyase activity

Concentrations of H_2_O_2 _in the medium were detected by use of the Amplex Red Hydrogen Peroxide/Peroxidase Assay Kit (A22188) from Molecular Probes (Karlsruhe, Germany) according to the supplier information. Fluorescence of the Amplex Red reagent was measured at 590 ± 4 nm after excitation at 530 ± 4 nm. In medium of the desired pH (6, 7.5, and 9) specific calibrations were done and H_2_O_2 _values were calculated accordingly. Because of the membrane permeability of H_2_O_2 _the external concentrations were regarded to be in equilibrium with the internal concentrations. In order to exclude unspecific formation of H_2_O_2 _in the medium control experiments were performed by incubation of medium without cells under the same conditions. In order to eliminate H_2_O_2 _produced in cell cultures purified catalase enzyme of *C. glutamicum *(kind gift of Roche Applied Science, Mannheim, Germany) was added to cultures.

The oxidative damage of proteins was analyzed using the OxyBlot™ Protein Oxidation Detection Kit (S7150) provided by Millipore. Basically, carbonylations of protein side chains are regarded as marker for oxidative stress and the occurrence of reactive oxygen species. Total proteins were extracted from cells grown at pH 6, 7.5 and 9 and carbonyl groups were derivatized to 2,4-dinitrophenylhydrazone which can be detected by a specific antibody. The cystathionine lyase activity was measured in cell extracts of *C. glutamicum *cells grown at different pH values as described previously [50]. All measures were performed at least in triplicate and standard deviations were calculated or representative results are shown.

### Transcriptome analysis

Total RNA isolation (including cell harvest and lysis), cDNA synthesis, and array hybridisation were performed as described previously [16], using 70 mer oligo microarrays instead of dsDNA microarrays. Spot finding, signal background segmentation and intensity quantification were carried out with the ImaGene 6.0 software (BioDiscovery). Normalization using the lowess function, which computes the logarithmic intensity ratio (m) and the logarithmic mean signal intensity (a) for each spot was performed and t-test statistics was accomplished with the EMMA microarray data analysis software [51]. Evaluation of the hybridization experiment was done as described in [23], using a m-value cut-off of ± 1, which corresponds to expression changes equal or greater than twofold. Since Hüser et al. (2003) found that an m-value cutoff of ± 0.6 equals a false-positive rate of 1%, at ± 1 this rate is 0.04% (roughly one false-positive among 3000 genes). The microarray data are available at the public repository ArrayExpress http://www.ebi.ac.uk/arrayexpress by the accession number E-MTAB-151.

### Proteome analysis

*C. glutamicum *ATCC13032 cells were harvested by centrifugation for 15 min at 4500 × g; cells were washed (PBS, 137 mM NaCl, 2.7 mM KCl, 10 mM Na_2_HPO_4_, 1.8 mM KH_2_PO_4_; pH 7.4) and disintegrated (PBS containing additional 20 mM MgCl_2_, 10 mM MnCl_2_, 200 U/ml DNaseI, protease inhibitor mix for bacterial cells (Sigma, St. Louis, MO, USA)) by four French press treatments (20000 psi, Thermo Spectronic, Rochester, USA). After centrifugation (5000 × g, 4°C) fractionation of the supernatant was performed. The soluble protein fraction was obtained as the supernatant after ultracentrifugation (100,000 × g; 4°C; 35 min). The membrane fraction was obtained after repeated ultracentrifugation and resuspension of the pellet in PBS with 10% glycerol and protease inhibitors. All samples were stored at -80°C.

For all approaches two technical replicates were performed. After inactivation of the protease inhibitor (60°C; 1 h) 100 μg of soluble proteins were incubated over night at 60°C with 4 μg trypsin (Promega, Madison, USA) and samples were desalted by Spec PT C18 AR solid phase extraction pipette tips (Varian, Lake Forest, CA, USA). The membrane fraction was treated according to two different protocols: (a) the enriched membrane fraction whereas a predigest removes membrane-associated proteins was achieved by the SIMPLE (Specific Integral Membrane Peptide Level Enrichment) protocol [52]; (b) the cell envelope fraction was achieved by a direct tryptic digest [53]. After removal of membranes by centrifugation (100,000 × g; 4°C; 35 min) samples were desalted with Spec PT C18 AR tips.

All desalted samples were resuspended in buffer A (2% acetonitrile, 0.1% formic acid) and subjected to 1D-nLC-ESI-MS using an autosampler. A self-packed capillary column was used for LC (Eclipse C18-RP XDB, Hewlett Packard) in combination with the Accela gradient HPLC pump system (Thermo Electron) coupled to an LTQ Orbitrap mass spectrometer (Thermo Electron). For elution of the peptides a multiple step gradient of buffer A to buffer B (80% acetonitril, 0.1% formic acid) was applied (0-5 min: 0% buffer B; 5-10 min: 10% buffer B; 10-175 min: 40% buffer B; 175-200 min: 100% buffer B; 200-210 min: 0% buffer B) at a flow rate of ~250 nl/min and a spray voltage of 1.5-1.8 kV. The LTQ Orbitrap was operated *via *instrument method files of Xcalibur (Rev. 2.0.7). The linear ion trap and orbitrap were operated in parallel, i.e. during a full MS scan on the orbitrap in the range of 300-2000 m/z at a resolution of 60,000, MS/MS spectra of the four most intense precursors were detected in the ion trap. Singly charged and more than triply charged ions were rejected from MS/MS and dynamic exclusion was enabled.

All database searches were performed using SEQUEST algorithm, embedded in Bioworks™ (Rev. 3.3, Thermo Electron), according the *Corynebacterium glutamicum *ATCC 13032 Bielefeld database [8]. The mass tolerance for precursor ions was set to 10 ppm; the mass tolerance for fragment ions was set to 1 amu. For protein identification a threshold for both protein and peptide probability was set to 0.001 in Bioworks™. MS^2 ^spectra per protein were counted using an in-house Perl script from Bioworks™ result tables. Spectral counts [17], i.e. number of identified peptide MS spectra per protein of different samples were normalized according the sum of all spectra in the sample. The significance of protein abundance changes was calculated in relation to the total peptide counts for each protein [54]. The proteome data are available at the public repository PRIDE at http://www.ebi.ac.uk/pride/ by the accession numbers 9355-9390.

### Metabolome analysis

Cell disruption and metabolite extraction was performed as described previously [55,56]. Derivatisation of samples as well as GC-MS analysis using a TraceGC gas chromatograph equipped with an AS2000 auto sampler and coupled to a PolarisQ ion trap mass spectrometer (Thermo Finnigan, Dreieich, Germany) was performed according to previous analyses [56]. The t-test algorithm of Excel 2000 (Microsoft, Seattle, WA) was used for determining whether observations were significantly different (P < 0.05).

## Authors' contributions

MF performed the analysis of the internal pH and bioenergetic parameters, IO performed the enzyme assays and the determination of H_2_O_2_, DS performed the OxyBlot assay, CT and AP performed the proteome analysis, AH and CR performed the DNA microarray analyses, MP performed the metabolome analysis, JK supervised the transcriptome and metabolome analysis. KM and RK designed the research and wrote the manuscript with assistance by CR, CT and JK.

## Supplementary Material

Additional file 1 **Exclusive alterations at the protein level at pH 6**. Table of proteins for which a differential peptide number was found at pH 6 in comparison to pH 7.5 but no alteration of the mRNA level was observed. footnotes for Table. ^1 ^The geneID according to the accession number BX927147 was used. ^2 ^Prediction of transmembrane helices were performed by using the TMHMM 2.0 sever at http://www.cbs.dtu.dk/services/TMHMM/. ^3 ^The induction factors are given as log_2 _values of the ration of mRNA levels at pH 6 and pH 9 in comparison to pH 7.5, respectively. ^4 ^The determined relative peptide numbers are given as log_2 _values in order to allow calculation of ratios by simple subtraction of values. Peptide numbers found to be significantly altered at pH 6 and pH 9 in comparison to pH 7.5 are shown in bold and peptide numbers found to be significantly altered at pH 6 in comparison to pH 9 are shown in italic (see M&M section for the details of calculation). Click here for file

Additional file 2 **Exclusive alterations at the protein level at pH 9**. Table of proteins for which a differential peptide number was found at pH 9 in comparison to pH 7.5 but no alteration of the mRNA level was observed. footnotes for Table. ^1 ^The geneID according to the accession number BX927147 was used. ^2 ^Prediction of transmembrane helices were performed by using the TMHMM 2.0 sever at http://www.cbs.dtu.dk/services/TMHMM/. ^3 ^The induction factors are given as log_2 _values of the ration of mRNA levels at pH 6 and pH 9 in comparison to pH 7.5, respectively. ^4 ^The determined relative peptide numbers are given as log_2 _values in order to allow calculation of ratios by simple subtraction of values. Peptide numbers found to be significantly altered at pH 6 and pH 9 in comparison to pH 7.5 are shown in bold and peptide numbers found to be significantly altered at pH 6 in comparison to pH 9 are shown in italic (see M&M section for the details of calculation). Click here for file

Additional file 3 **Analysis of protein modifications by oxidative stress using the detection of carbonyl groups in protein side chains**. Total protein extracts of cells grown at pH 6, 7.5 and 9 were obtained and subjected to an 1D SDS-PAGE before (A) and after the derivatization by 2,4-dinitrophenylhydrazine (DNP, B). The DNP mojety was detected using a specific antibody of the OxyBlot detection kit. Click here for file
