# Supplementary material for: Functional genomics of pH homeostasis in Corynebacterium glutamicum revealed novel links between pH response, oxidative stress, iron homeostasis and methionine synthesis
Source: BMC Genomics. 2009 Dec 21;10:621. doi: 10.1186/1471-2164-10-621 (PMC2807442; doi:10.1186/1471-2164-10-621)
Supplement: Additional file 1 — Exclusive alterations at the protein level at pH 6. Table of proteins for which a differential peptide number was found at pH 6 in comparison to pH 7.5 but no alteration of the mRNA level was observed. footnotes for Table. 1 The geneID according to the accession number BX927147 was used. 2 Prediction of transmembrane helices were performed by using the TMHMM 2.0 sever at http://www.cbs.dtu.dk/services/TMHMM/. 3 The induction factors are given as log2 values of the ration of mRNA levels at pH 6 and pH 9 in comparison to pH 7.5, respectively. 4 The determined relative peptide numbers are given as log2 values in order to allow calculation of ratios by simple subtraction of values. Peptide numbers found to be significantly altered at pH 6 and pH 9 in comparison to pH 7.5 are shown in bold and peptide numbers found to be significantly altered at pH 6 in comparison to pH 9 are shown in italic (see M&M section for the details of calculation). [file 1471-2164-10-621-S1.PDF]

Proteins induced at pH 6

					Transcriptome[3]		Proteome[4]								
							cytoplasma			envelop			membrane		
**No**	**gene ID[1]**	**gene**	**function**	**TMH[2]**	**pH 6**	**pH 9**	**6**	**7.5**	**9**	**6**	**7.5**	**9**	**6**	**7.5**	**9**
1	*cg0044*		ABC-type transporter, periplasmic component	0	-0.28	-	-	-	-	0.8	-0.2	1.5	***5.6***	4.4	*4.1*
2	*cg0286*		Conserved hypothetical protein	6	-	-	-	-	-	-	-	-	3.9	2.2	-0.4
3	*cg0336*	*ponA*	Membrane carboxypeptidase	1	-0.04	-	-	-	-	3.8	2.7	3.2	4.3	0.4	2.7
4	*cg0414*	*wzz*	ATPases involved in chromosome partitioning	2	-0.34	-0.66	-	-0.8	-	3.9	4.1	3.8	4.4	3.4	3.2
5	*cg0683*		Hypothetical transport protein	9	0.97	-0.87	-	-	-	4.4	0.7	4.4	5.0	3.9	4.4
6	*cg0735*		ABC transporter, transmembrane component	5	-	-	-	-	-	1.7	-0.2	0.0	4.3	2.0	-
7	*cg0771*	*irp1*	ABC-type transporter, periplasmic component	0	-0.36	-	3.8	3.8	-0.8	4.2	3.2	-0.1	**5.4**	3.4	1.6
8	*cg0812*	*dtsR*	Detergent sensitivity rescuer	1	0.95	-0.23	2.8	2.8	2.6	***5.2***	3.7	*3.8*	1.9	2.0	0.5
9	*cg1001*	*mscL*	Large-conductance mechanosensitive channel	2	0.09	-0.52	0.6	0.4	-	4.1	2.9	1.9	4.3	3.0	1.5
10	*cg1111*	*eno*	Enolase	0	0.24	0.09	***8.4***	7.8	***6.7***	2.5	-0.2	1.4	1.4	-	-0.6
11	*cg1228*		ABC-type transporter, duplicated ATPase component	0	0.16	0.15	3.3	4.1	3.6	**7.8**	7.2	**7.7**	6.1	5.8	6.0
12	*cg1290*	*metE*	Homocysteine methyltransferase	0	0.65	-1.06	***8.2***	6.8	*6.4*	*8.0*	8.0	***6.5***	*6.2*	6.4	***4.2***
13	*cg1451*	*serA*	Phosphoglycerate dehydrogenase	0	-0.22	-1.1	**6.5**	5.4	6.1	*7.7*	7.5	*7.2*	*5.3*	5.3	***4.2***
14	*cg1556*		Hypothetical protein	0	-0.07	0.01	2.0	3.1	0.3	***5.6***	4.1	*4.6*	2.8	1.6	1.6
15	*cg1604*		Hypothetical protein	1	-	-0.15	1.2	2.1	2.3	*2.8*	3.1	***5.4***	3.9	-0.6	2.0
16	*cg1624*		NhaP-type Na+/H+ antiporter	11	0.51	-0.44	-	-	-	3.2	-0.2	1.4	3.7	0.7	-0.4
17	*cg1656*	*ndh*	NADH dehydrogenase, FAD-containing subunit	1	-0.09	-0.23	***5.9***	6.6	***4.4***	***8.2***	7.5	*7.7*	***6.6***	5.5	*5.6*
18	*cg1786*	*secG*	Protein-export membrane protein	2	-0.02	-0.09	-	-	-	0.9	0.7	1.4	4.1	-0.6	1.4
19	*cg2155*		Hypothetical protein	0	0.32	-0.55	**4.7**	2.9	3.6	-	-	-	-	-	-
20	*cg2299*	*hisA*	Imidazole-4-carboxamideisomerase	0	0.33	-0.28	3.9	2.6	3.8	-	-	-	-	-	-
21	*cg2424*		Hypothetical membrane protein	2	-	-0.23	-	-	-	5.0	4.2	4.8	***4.4***	2.1	*2.6*
22	*cg2613*	*mdh*	Malate dehydrogenase	0	0.31	0.21	**4.7**	3.4	4.6	-	-	-	-0.9	-	-0.6
23	*cg2799*	*pknE*	Putative secreted protein	1	-0.27	-0.38	-	-	-	3.2	4.4	4.0	***5.0***	3.5	*3.0*
24	*cg2888*	*cgtR*	Two-component system, response regulator	0	-0.21	0.82	3.9	2.1	4.2	-	-	0.7	-	-	-
25	*cg2958*	*butA*	L-2.3-butanediol dehydrogenase	0	0.74	0.22	**5.5**	4.2	4.9	5.5	5.9	5.2	3.9	3.6	1.6
26	*cg3019*		Putative secreted protein	2	-	-	-	-	-	3.3	3.2	3.0	***4.7***	3.0	*2.3*
27	*cg3138*		Membrane protease subunits	2	-	0.65	-	0.2	-	3.8	2.1	5.0	**5.3**	3.8	**6.0**
28	*cg3244*		Hypothetical protein	0	-0.28	-0.24	-0.3	2.0	-	3.7	2.3	2.8	3.8	2.0	2.7
29	*cg3301*		Permease of the major facilitator superfamily	12	0.14	-	-	-	-	1.7	0.8	0.9	4.2	2.1	-0.4
30	*cg3313*	*mrcB*	Membrane carboxypeptidase	1	-0.39	-0.29	-	-	-	3.9	2.8	3.9	3.0	1.2	1.8
31	*cg3366*	*rmpA*	PTS system mannitol/fructose-specific IIA domain	0	0.74	-0.85	1.9	-0.8	-	4.3	2.8	0.9	**5.0**	1.8	0.5

Proteins repressed at pH 6

					Transcriptome[3]		Proteome[4]								
							cytoplasma			envelop			membrane		
**No**	**gene ID[1]**	**gene**	**function**	**TMH[2]**	**pH 6**	**pH 9**	**6**	**7.5**	**9**	**6**	**7.5**	**9**	**6**	**7.5**	**9**
1	*cg0133*		P-aminobenzoyl-glutamate transporter	13	-0.86	-1.02	-	-	-	-	-	-	0.3	3.7	2.1
2	*cg0468*		Cobalamin/Fe3+-siderophores transport system	8	-0.79	-	-	-	-	-	0.8	-	2.8	4.3	1.1
3	*cg0592*		Acetyl-CoA hydrolase	0	-	-	1.1	3.8	3.2	**2.2**	5.2	**1.5**	-	-	-
4	*cg0674*	*rpsI*	30S ribosomal protein S9	0	-0.92	-0.28	**2.8**	4.4	3.1	-0.4	-	0.0	-0.7	-0.6	-
5	*cg0759*	*prpD*	2-methylcitrate dehydratase 2	0	-	-	-0.6	2.6	2.0	0.6	4.4	-	-	-0.6	-
6	*cg0989*	*rpsN*	30S ribosomal protein S14	0	-0.71	-0.25	2.5	3.9	1.7	0.0	0.7	2.7	1.2	1.0	1.2
7	*cg1333*	*argS*	Arginyl-tRNA synthetase	0	-0.51	-0.17	1.9	2.9	2.2	6.3	6.8	**5.9**	0.1	3.1	-0.6
8	*cg1726*	*mcmA*	Methylmalonyl-CoA mutase	0	-0.53	-0.79	1.3	3.3	3.1	**2.9**	4.5	3.2	1.8	3.7	2.1
9	*cg1859*		Putative secreted protein	0	0.04	-1.34	-	1.7	-	-	3.6	1.5	0.1	5.0	-0.4
10	*cg2184*		ABC-type transporter, duplicated ATPase component	0	-	-0.47	-	-0.5	-	**2.1**	4.5	4.2	-	4.2	2.7
11	*cg2195*		Putative secreted or membrane protein	2	0.24	0.11	-	-	-	-	1.3	2.1	2.3	4.1	3.3
12	*cg2257*	*srp*	Signal recognition particle GTPase	0	0.43	0.01	1.9	4.1	1.4	2.2	1.8	1.0	-0.9	-	-
13	*cg2325*		Hypothetical protein	0	-0.46	0.3	-	-	-	-	-	-	-0.9	4.7	3.8
14	*cg2833*	*cysK*	O-Acetylserine (Thiol)-Lyase	0	0.42	-0.14	6.9	6.7	6.5	**4.5**	6.0	**4.3**	*4.8*	4.1	*2.5*
15	*cg3008*	*porA*	Porin	0	0.05	-0.31	-	-	-	4.2	5.2	4.3	**6.7**	7.7	**6.4**
16	*cg3009*	*porH*	Hypothetical protein	0	0.09	-0.1	-	-	-	-	-	-	**5.3**	6.4	**5.2**
17	*cg3018*		Hypothetical protein	0	0.14	0.2	0.7	2.4	1.1	4.5	4.9	**3.6**	1.2	1.7	-0.6
