# Supplementary material for: Functional genomics of pH homeostasis in Corynebacterium glutamicum revealed novel links between pH response, oxidative stress, iron homeostasis and methionine synthesis
Source: BMC Genomics. 2009 Dec 21;10:621. doi: 10.1186/1471-2164-10-621 (PMC2807442; doi:10.1186/1471-2164-10-621)
Supplement: Additional file 2 — Exclusive alterations at the protein level at pH 9. Table of proteins for which a differential peptide number was found at pH 9 in comparison to pH 7.5 but no alteration of the mRNA level was observed. footnotes for Table. 1 The geneID according to the accession number BX927147 was used. 2 Prediction of transmembrane helices were performed by using the TMHMM 2.0 sever at http://www.cbs.dtu.dk/services/TMHMM/. 3 The induction factors are given as log2 values of the ration of mRNA levels at pH 6 and pH 9 in comparison to pH 7.5, respectively. 4 The determined relative peptide numbers are given as log2 values in order to allow calculation of ratios by simple subtraction of values. Peptide numbers found to be significantly altered at pH 6 and pH 9 in comparison to pH 7.5 are shown in bold and peptide numbers found to be significantly altered at pH 6 in comparison to pH 9 are shown in italic (see M&M section for the details of calculation). [file 1471-2164-10-621-S2.PDF]

Proteins induced at pH 9

No	gene ID[1]	gene	function	TMH[2]	Transcriptome[3]		Proteome[4]								
							cytoplasma			envelop			membrane		
**No**	**gene ID[1]**	**gene**	**function**	**TMH[2]**	**pH 6**	**pH 9**	**6**	**7.5**	**9**	**6**	**7.5**	**9**	**6**	**7.5**	**9**
1	*cg0422*	*murA*	UDP-N-acetylglucosamine enolpyruvyl transferase	0	-0.46	-0.4	2.9	2.6	3.8	2.6	2.3	0.8	-	1.9	-
2	*cg0673*	*rplM*	50S ribosomal protein L13	0	-0.84	-0.32	-	-0.8	1.3	2.6	2.3	3.8	-0.9	1.0	2.9
3	*cg0766*	*icd*	Isocitrate dehydrogenase	0	-0.06	0.09	*5.4*	5.1	***6.5***	3.2	3.8	3.3	1.5	2.2	1.0
4	*cg0873*	*aroA*	3-phosphoshikimate 1-carboxyvinyltransferase	0	0.25	0.96	2.2	2.1	3.9	-	-	-	-	-	-
5	*cg1408*		Putative membrane protein	2	-0.14	0.64	-0.3	-0.5	-	**5.0**	3.5	**5.5**	0.1	-	-
6	*cg1604*		Hypothetical protein	1	-	-0.15	1.2	2.1	2.3	*2.8*	3.1	***5.4***	3.9	-0.6	2.0
7	*cg1730*		Membrane protease subunits	1	0.06	-0.43	1.0	-	-0.8	2.7	2.5	4.2	1.8	0.4	1.9
8	*cg1769*	*ctaA*	Cytochrome oxidase assembly protein	8	-	-	-	-	-	0.7	0.7	3.7	3.6	3.0	4.1
9	*cg1787*	*ppc*	Phosphoenolpyruvate carboxylase	0	0.36	-	2.4	2.4	4.1	5.7	5.5	5.8	2.3	3.6	4.0
10	*cg2299*	*hisA*	Imidazole-4-carboxamideisomerase	0	0.33	-0.28	3.9	2.6	3.8	-	-	-	-	-	-
11	*cg2405*	*qcrC*	Menaquinol-cytochrome c reductase subunit	2	-	0.23	-	-	-	4.4	4.8	5.4	3.8	2.4	3.6
12	*cg2523*	*malQ*	4-alpha-glucanotransferase	0	0.27	-0.01	3.7	2.8	3.9	5.2	5.6	**3.6**	2.3	2.2	1.5
13	*cg2613*	*mdh*	Malate dehydrogenase	0	0.31	0.21	4.7	3.4	4.6	-	-	-	-0.9	-	-0.6
14	*cg2750*		Hypothetical membrane protein	2	0.62	0.96	-	-	-	3.2	2.1	4.0	1.4	2.0	3.2
15	*cg2780*	*ctaD*	Cytochrome c oxidase polypeptide I	12	0.31	0.52	-	-	-	3.7	2.1	3.9	*5.1*	5.3	***6.3***
16	*cg2888*	*cgtR*	Two-component system, response regulator	0	-0.21	0.82	3.9	2.1	4.2	-	-	0.7	-	-	-
17	*cg3138*		Membrane protease subunits	2	-	0.65	-	0.2	-	3.8	2.1	**5.0**	**5.3**	3.8	**6.0**
18	*cg3179*	*fadD*	Putative Acyl-CoA synthetase	0	0.18	0.6	1.1	3.2	4.1	1.8	0.8	3.7	-	-	-
19	*cg3219*	*ldh*	L-lactate dehydrogenase	0	1.83	0.12	**5.1**	3.1	**4.8**	4.3	1.3	2.3	-	-	-0.4
20	*cg3315*		Transcriptional regulator	0	0.03	0.71	3.6	3.2	4.5	-0.4	-	-	-	-	-0.6
21	*cg3327*	*dps*	Starvation-inducible DNA-binding protein	0	0.32	0.75	1.6	-0.8	3.6	-	-	-	-	-	-

Proteins repressed at pH 9

No	gene ID[1]	gene	function	TMH[2]	Transcriptome[3]		Proteome[4]								
							cytoplasma			envelop			membrane		
**No**	**gene ID[1]**	**gene**	**function**	**TMH[2]**	**pH 6**	**pH 9**	**6**	**7.5**	**9**	**6**	**7.5**	**9**	**6**	**7.5**	**9**
1	*cg0007*	*gyrB*	DNA Gyrase subunit B	0	-0.7	-0.56	1.2	1.9	-0.8	4.2	4.5	3.3	0.2	2.0	-0.4
2	*cg0441*	*lpd*	Dihydrolipoamide dehydrogenase	0	-0.2	-0.04	4.1	4.3	0.3	0.6	-	-	0.8	-	-
3	*cg0468*		Cobalamin/Fe3+-siderophores transport system	8	-0.79	#NV	-	-	-	-	0.8	-	2.8	4.3	1.1
4	*cg0628*	*rpsH*	30S ribosomal protein S8	0	-0.48	-0.19	4.5	4.9	3.8	2.2	2.1	4.1	3.7	4.4	4.5
5	*cg0674*	*rpsI*	30S ribosomal protein S9	0	-0.92	-0.28	2.8	4.4	3.1	-0.4	-	0.0	-0.7	-0.6	-
6	*cg0752*		Uncharacterized BCR	1	-1.55	-0.47	0.6	2.7	1.6	3.4	2.3	1.8	4.4	4.7	3.4
7	*cg0989*	*rpsN*	30S ribosomal protein S14	0	-0.71	-0.25	2.5	3.9	1.7	0.0	0.7	2.7	1.2	1.0	1.2
8	*cg1111*	*eno*	Enolase	0	0.24	0.09	***8.4***	7.8	***6.7***	2.5	-0.2	1.4	1.4	-	-0.6
9	*cg1314*	*putP*	Na+/proline symporter	13	-0.55	-0.04	-	-	-	3.3	3.8	3.0	4.4	4.5	3.1
10	*cg1322*		Uncharacterized BCR	0	1.24	-0.62	***6.3***	5.1	***2.7***	-	-	-	2.6	-0.6	-
11	*cg1333*	*argS*	Arginyl-tRNA synthetase	0	-0.51	-0.17	1.9	2.9	2.2	6.3	6.8	**5.9**	0.1	3.1	-0.6
12	*cg1364*	*atpF*	F0F1-type ATP synthase b subunit	1	-1.52	-0.96	-0.6	0.5	-0.8	4.5	4.8	4.9	4.9	5.4	**4.3**
13	*cg2132*		Hypothetical protein	0	#NV	-0.35	*4.9*	5.1	***3.1***	-	-	-	-0.9	-	-
14	*cg2833*	*cysK*	O-Acetylserine (Thiol)-Lyase	0	0.42	-0.14	6.9	6.7	6.5	**4.5**	6.0	**4.3**	*4.8*	4.1	*2.5*
15	*cg3018*		Hypothetical protein	0	0.14	0.2	0.7	2.4	1.1	4.5	4.9	**3.6**	1.2	1.7	-0.6
