# Supplementary figures and images for: Functional genomics of pH homeostasis in Corynebacterium glutamicum revealed novel links between pH response, oxidative stress, iron homeostasis and methionine synthesis
Source: BMC Genomics. 2009 Dec 21;10:621. doi: 10.1186/1471-2164-10-621 (PMC2807442; doi:10.1186/1471-2164-10-621)

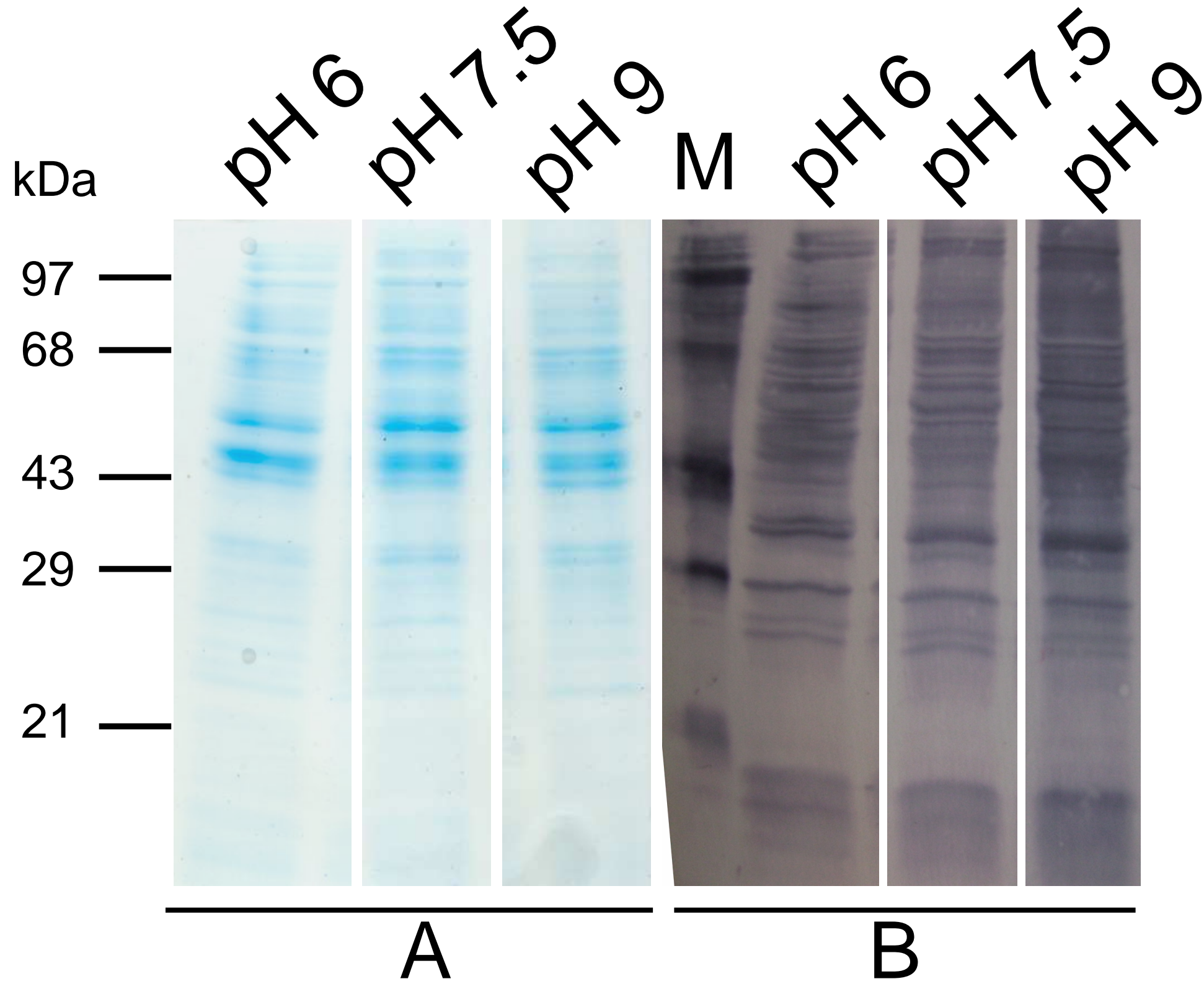

Supplement: Additional file 3 — Analysis of protein modifications by oxidative stress using the detection of carbonyl groups in protein side chains. Total protein extracts of cells grown at pH 6, 7.5 and 9 were obtained and subjected to an 1D SDS-PAGE before (A) and after the derivatization by 2,4-dinitrophenylhydrazine (DNP, B). The DNP mojety was detected using a specific antibody of the OxyBlot detection kit. [file 1471-2164-10-621-S3.PDF]
